# Comprehensive transcript profiling of two grapevine rootstock genotypes contrasting in drought susceptibility links the phenylpropanoid pathway to enhanced tolerance

**DOI:** 10.1093/jxb/erv274

**Published:** 2015-06-02

**Authors:** Massimiliano Corso, Alessandro Vannozzi, Elie Maza, Nicola Vitulo, Franco Meggio, Andrea Pitacco, Andrea Telatin, Michela D’Angelo, Erika Feltrin, Alfredo Simone Negri, Bhakti Prinsi, Giorgio Valle, Angelo Ramina, Mondher Bouzayen, Claudio Bonghi, Margherita Lucchin

**Affiliations:** ^1^Department of Agronomy, Food, Natural resources, Animals and Environment (DAFNAE), University of Padova Agripolis, 35020 Legnaro, Italy; ^2^Centro Interdipartimentale per la Ricerca in Viticoltura ed Enologia (CIRVE), Via XXVIII Aprile, 14-31015 Conegliano (TV), Italy; ^3^Genomics and Biotechnology of Fruit (GBF) Laboratory, Institut National Polytechnique de Toulouse, Avenue de l’Agrobiopole, F-31326 Castanet-Tolosan Cedex (Toulouse), France; ^4^CRIBI, University of Padova, viale G. Colombo 3, 35121 Padova, Italy.; ^5^Department of Agricultural and Environmental Sciences-Production, Landscape, Agroenergy (DiSAA), University of Milano, Milano 20133, Italy

**Keywords:** Flavonoids, genome re-sequencing, mRNA-Seq, stilbenes, *Vitis*, water stress.

## Abstract

In light of ongoing climate changes in wine-growing regions, the selection of drought-tolerant rootstocks is becoming a crucial factor for developing a sustainable viticulture. In this study, M4, a new rootstock genotype that shows tolerance to drought, was compared from a genomic and transcriptomic point of view with the less drought-tolerant genotype 101.14. The root and leaf transcriptome of both 101.14 and the M4 rootstock genotype was analysed, following exposure to progressive drought conditions. Multifactorial analyses indicated that stress treatment represents the main factor driving differential gene expression in roots, whereas in leaves the genotype is the prominent factor. Upon stress, M4 roots and leaves showed a higher induction of resveratrol and flavonoid biosynthetic genes, respectively. The higher expression of *VvSTS* genes in M4, confirmed by the accumulation of higher levels of resveratrol in M4 roots compared with 101.14, was coupled to an up-regulation of several *VvWRKY* transcription factors. Interestingly, *VvSTS* promoter analyses performed on both the resequenced genomes highlighted a significantly higher number of W-BOX elements in the tolerant genotype. It is proposed that the elevated synthesis of resveratrol in M4 roots upon water stress could enhance the plant’s ability to cope with the oxidative stress usually associated with water deficit.

## Highlights

Drought tolerance in the M4 grapevine rootstock genotype could be associated with a higher capability to counteract oxidative stresses by enhancing the accumulation of resveratrol in roots.

## Introduction

Modern viticulture is almost exclusively based on the use of scions grafted onto interspecific rootstocks. This widespread agronomical practice is based on the fact that grapevine rootstocks are not only able to confer resistance to various root pathogens, but also impart a large range of advantages by altering numerous physiological processes at the scion level, such as biomass accumulation (Gregory *et al.*, 2013), fruit quality (Walker *et al.*, 2002), and the ability to respond to many abiotic stresses (Fisarakis *et al.*, 2001; Marguerit *et al.*, 2012; Covarrubias and Rombolà, 2013; Meggio *et al.*, 2014). All of these characteristics make the use of rootstocks and the development of new rootstock genotypes of crucial importance in contemporary viticulture.

Water availability is one of the major environmental factors limiting viticultural production (Chaves *et al.*, 2010). Most wine-producing regions in the world are subjected to seasonal drought, and, based on the global climate models predicting an increase in aridity in the future, water deficit is likely to become the major limiting factor in wine production and quality. Generally, drought is associated with many morphological and physiological changes in plants over a range of spatial and temporal scales (Chaves *et al.*, 2002), including reduced expansion of aerial organs (Cramer *et al.*, 2007), decrease in transpiration and photosynthesis (Chaves *et al.*, 2009, 2010), accumulation of osmotic compounds and ions (Cramer *et al.*, 2007), activation of detoxifying processes, and, in parallel, the transcriptional regulation of a large number of genes (Cramer *et al.*, 2007; Tillett *et al.*, 2011). Also root development is negatively influenced by drought stress, although to a lesser extent than that of the shoot. The different sensitivity to water scarcity shown by the two organs results in a decrease in the shoot:root mass ratio, as observed in many plants upon water stress (WS) (Blum *et al.*, 1996).

Grapevines are well adapted to semi-arid climates such as that of Mediterranean regions and are generally considered to be relatively tolerant to water deficit. Their large and deep root systems, together with physiological drought avoidance mechanisms, such as stomatal control of transpiration, xylem embolism, and the ability to adjust osmotically, mean that these plants are able to remain productive under suboptimal water regimes (Lovisolo *et al.*, 2002). However, with a large proportion of vineyards located in regions where seasonal drought coincides with the grapevine growing season, the combined effects of soil water deficit, air temperature, and high evaporative demand are known to limit yield and delay the vintage date (Chaves *et al.*, 2009, 2010; Flexas *et al.*, 2009), with a negative effect on the berries and, consequently, wine quality.

The grapevine photosynthetic process is reasonably resistant to WS (De Souza *et al.*, 2003; Chaves *et al.*, 2009). However, as the stress becomes severe, net CO_2_ assimilation and other metabolic processes operating in the mesophyll are inhibited, and water use efficiency declines. The imbalance between light capture and its utilization results in changes in the photochemistry of chloroplasts with the generation of reactive oxygen species (ROS), such as H_2_O_2_, O_2_^–^, –OH, and RO_2_, and nitric oxide (NO) responsible for most of the oxidative damage in biological systems and cellular components (Apel and Hirt, 2004; Kar, 2011).

Phytohormones play a central role in the ability of plants to adapt to abiotic stresses by mediating a wide range of responses (Santner and Estelle, 2009). Amongst these, abscisic acid (ABA) is probably the most studied stress-responsive hormone in plants, because of its central role in regulating the plant response to water deficit (Fujita *et al.*, 2011; Qin *et al.*, 2011). ABA synthesis and accumulation is one of the earliest plant responses to abiotic stress, triggering ABA-induced gene expression and inducing stomatal closure to reduce water loss, and eventually limiting cellular growth (Peleg and Blumwald, 2011; Lata and Prasad, 2011). Although ABA is the most comprehensively studied hormone involved in the plant response to drought, a growing number of studies have revealed that many other hormones, including auxins, ethylene, jasmonates (JAs), gibberellins (GAs), salicylic acid (SA), and brassinosteroids (BRs), are also involved in response to stress (Peleg and Blumwald, 2011). Thus, the adaptation of plants to water-limiting conditions involves the concerted action of all of these hormones through fine-tuned cross-talk (Kohli *et al.*, 2013). Phytohormones also appear to cross-talk with ROS, which probably act as secondary messengers of these regulators (Kar, 2011). It is well known that H_2_O_2_ regulates ABA-mediated stomatal closure by acting on Ca^2+^ levels and inactivating protein phosphatase 2C (Meinhard *et al.*, 2002). Stomatal closure is also mediated by ethylene via ETR1, one of its receptors, which is involved in H_2_O_2_ sensing (Desikan *et al.*, 2005). Lastly, Joo *et al.* (2001) reported that ROS may function as a downstream component in the auxin-mediated signal in gravistimulated or auxin-treated maize roots.

A biochemical and physiological study of a novel genotype proposed to be used as rootstock in grapevine production was recently performed by Meggio *et al.* (2014). This genotype, named M4 [(*Vitis vinifera*×*V. berlandieri*)×*V. berlandieri*×cv Resseguier n. 1], was selected for its high tolerance to water deficit (WS) and salt exposure [salt stress (SS)]. When compared with the commercial genotype 101.14 (*V. riparia×V. rupestris*), M4 ungrafted plants showed a greater capacity to tolerate WS and SS, maintaining photosynthetic activity while under severe stress conditions. Here a large-scale whole-transcriptome analyses performed on leaf and root tissues of both M4 and 101.14 genotypes under the same WS experimental conditions as described previously by Meggio *et al.* (2014) is reported It is important to note that in contrast to previous drought studies in which plants were subjected to an immediate reduction in water availability, in the present experiment, water deprivation was accomplished gradually, mimicking conditions occurring in the field. Another innovative aspect of this study relies on the transcriptome comparative approach between a drought-tolerant and susceptible genotype with regard to WS, which has not been explored to date in the *Vitis* genus.

## Materials and methods

### Rootstock genotypes and genome resequencing

Grapevine rootstocks used in this study were the common drought-susceptible genotype 101.14 (*V. riparia×V. rupestris*) and the drought-tolerant genotype M4 [(*V. vinifera×V. berlandieri*)×*V. berlandieri* cv. Resseguier n. 1], recently described and physiologically characterized by Meggio *et al.* (2014). As both of these rootstocks are interspecific hybrids, genome resequencing was carried out as a preliminary step critical for further analyses, in order to describe the variability amongst single nucleotide polymorphisms (SNPs) and gene predictions between 101.14, M1, and the reference PN40024 genome (Jaillon *et al.*, 2007). Briefly, genomic DNA was isolated from young leaves, and for each genotype a mate-pair DNA library was constructed, which was sequenced using the SOLiD 5500×l platform (Life Technologies), following the manufacturer’s instructions. Alignments of the 101.14 and M4 DNA sequences onto PN40024 were performed with PASS v. 2.0 software (Campagna *et al.*, 2009) using auto-optimized parameters for trimming and 80% identity. The same platform was used for SNP calling. *De novo* assembly of unaligned reads was obtained with an optimized script of the Velvet 1.2 package (VelvetOptimize; Zerbino and Birney, 2008) fixing a k-mer size of 27. Results are available at the http://genomes.cribi.unipd.it/grape/serres/ website.

### Experimental design

For each genotype, 72 two-year-old plants were glasshouse grown in 3 litre pots filled with sand–peat mixture (7:3 v/v) with a water content maintained to 80% of soil field capacity calculated gravimetrically by the difference in weight between the wet and the dry soil. Plants were divided into two groups: plants grown under well-watered (WW) conditions (control) and plants grown under water stress (WS) conditions. The WS was gradually imposed by decreasing the water availability in pots from 80% to 30% of field capacity, whereas WW plants, used as control, were maintained to 80% of field capacity (Supplementary Fig. S1 available at *JXB* online). The whole experiment lasted 10 d, during which four samplings, designated as T1–T4 and corresponding to 2, 4, 7, and 10 days after stress imposition (DASI), were performed for both leaves and roots.

### Leaf physiology measurements

During the experimental period, the leaf transpiration rate (*E*, mmol H_2_O m^–2^ s^–1^) was measured (between 11:00h and 14:00h solar time) on two fully expanded leaves per plant using a LI-6400 portable photosynthesis system (Li-Cor Inc., Lincoln, NE, USA) under a constant saturating photosynthetic photon flux density (PPFD) of 600 μmol of photons m^–2^ s^–1^, CO_2_ concentration of 380 μmol mol^−1^, block temperature of 25 °C, and relative humidity between 60% and 70% allowing ~1.5 kPa of vapour pressure deficit (VPD) inside the leaf chamber.

### mRNA-Seq and metabolite analyses

Leaves and roots of both 101.14 and M4 genotypes grown upon WW and WS conditions were collected from three plants at T1, T2, and T3 and from six plants at T4 and pooled as previously described in Meggio *et al.* (2014). For both genotypes, the zero time samples (T0) were collected from six plants of each genotype grown under WW conditions. The whole experiment was performed on two separate biological replicates for a total of 36 samples from leaves and 36 samples from roots [2 genotypes×2 treatments×4 time points (T1–T4)+2 genotypes in WW conditions (T0)=18 samples×2 replicates] (Supplementary Fig. S1 at *JXB* online). Methods for whole-transcriptome analysis are reported in Supplementary Methods S1. The mRNA sequencing (mRNA-Seq) data obtained in this study have been deposited at the NCBI Short Read Archive (http://www.ncbi.nlm.nih.gov/Traces/sra/sra.cgi) under accession number SRA110531.

ABA was extracted from leaf tissues using a modified protocol based on Zhang *et al.* (2008) (Supplementary Methods S2 at *JXB* online). For stilbene quantification, root samples were powdered in liquid N_2_ and extracted in 3 vols of 90% (v/v) methanol, 0.1% (v/v) formic acid (FA). After shaking at 4 °C for 10min, samples were centrifuged at 10 000 *g* for 10min at 4 °C, filtered by Millipore Millex HV cartridges (0.45 μm), and dried in a Speed-Vac at room temperature for 90min. The pellets were then solubilized in 4% (v/v) methanol, 0.1% (v/v) formic acid. Liquid chromatography-elecrospray ionization-mass spectrometry (LC-ESI-MS) analyses were conducted using an Agilent Technologies 1200 Series capillary pump coupled with a dual ESI source on a 6520 Q-TOF mass spectrometer. Briefly, LC runs were performed on an XDB-C18 column (2.1×50mm, 1.8 μm, Agilent Technologies) applying a 20min non-linear gradient of 0.1% (v/v) FA/acetonitrile, from 5% to 30%, with a flow rate of 200 μl min^–1^. The ESI source was set at 350 °C, drying gas (N_2_) at 5 l min^–1^, 3000V (positive mode), fragmentor at 75V, and the data acquisition range was 100–600 *m/z* at 2.03 scans s^–1^. The compound identification was conducted by extraction of the EIC for [MH+] (resveratrol, 229.09 *m/z*; piceid, 391.14 *m/z*) accepting a mass error of ±20 mDa and referring to calibration curves.

### Multifactorial and pairwise statistical analyses

Statistical analyses for discovering differentially expressed genes (DEGs) were performed with the *DEseq* R package (http://www.r-project.org/) (Maza *et al.*, 2013). In order to evaluate the individual effects of the genotype (101.14 and M4), treatment (WW and WS), and time point (T1–T4) on gene expression, a multifactorial analysis was conducted using the multifactor designs method of the *DEseq* R package (Anders and Huber, 2010; http://bioconductor.org/packages/release/bioc/html/DESeq.html). This method evaluates the weight of each factor considered in the analysis and its impact on DEGs, according to a false discovery rate (FDR)-adjusted *P*-value <0.05. The genotype effect (101.14 and M4) is indicated as ‘G’, the type of treatment imposed indicated as ‘T’ (WW and WS plants), and the time point considered within the stress treatment indicated as ‘P’ (T1, T2, T3, T4).

### Ontology and differential clustering analysis (DCA)

In order to classify those genes affected by WS treatment functionally, those DEGs that, based on the multifactorial analysis, were affected by all components (common DEGs between G, T, and P) and by the genotype and treatment (common DEGs between G and T), were associated with their Gene Ontology (GO) terms, imported in Blast2GO software v 2.5.0 and grouped into enriched GO categories (Götz *et al.*, 2008). Within the most represented GO categories, those DEGs associated with GO terms related to plant hormones, secondary metabolism, sugars, stresses, cell wall, and transcription factors were selected for a DCA. This approach, previously applied by Ihmels *et al.* (2005), was further improved to better capture differential expression patterns and systematically characterize both similarities and differences in the fine structure of co-regulation patterns. The modified version of the original DCA method and the R script are described in Supplementary Methods S3 at *JXB* online.

## Results

### The M4 genotype maintains higher transpiration rate compared with 101.14 upon drought

**Fig. 1.**
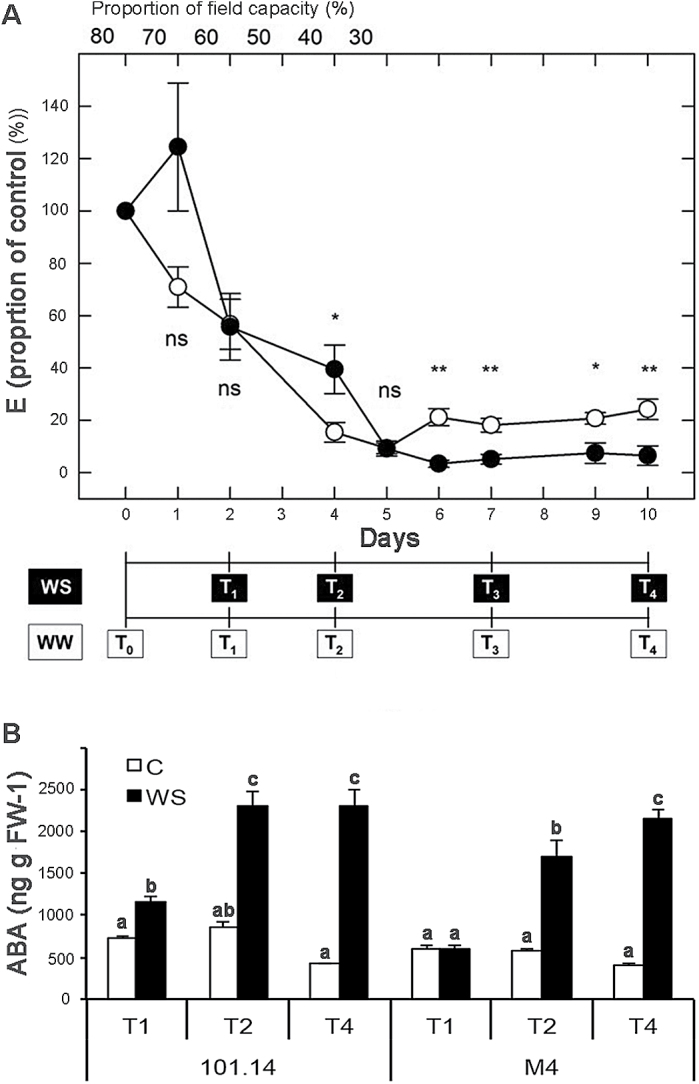
(A) Effect of water stress (WS) on leaf transpiration rate (*E*) of M4 (open circles) and 101.14 (filled circles) grapevine rootstocks. Average ±SE values of *E* are expressed as a proportion of the control (i.e. *E* values of 2.7±0.3 and 2.4±0.2 mmol H_2_O m^–2^ s^–1^ for M4 and 101.14 genotypes, respectively, at T0). Well-watered (WW) plants were maintained at 80% of soil field capacity. WS was induced by progressively reducing the soil water content down to 30% of field capacity. A field capacity of 30% by weight was obtained gravimetrically by the difference in weight between the wet and the dry soil. T1–T4 represent sampling times throughout the experimental period after control (T0). Significant differences amongst genotypes within a given time point are indicated as **(*P*< 0.01) and *(*P*<0.05) according to the Student’s *t*-test. (B) Leaf ABA content in 101.14 and M4 plants in both WW (empty bars) and WS conditions (solid bars). Measurements were carried out at T1, T2, and T4, corresponding to 2, 4, and 10 days after stress imposition (DASI), respectively. Letters indicate statistical differences in (two-tailed unpaired) Student’s *t*–test at *P*<0.01.

The response of the two rootstock genotypes used in this study (101.14 and M4) to WS in terms of net CO_2_ assimilation rate, stomatal conductance, leaf water potential, and tissue osmolality was previously reported by Meggio *et al.* (2014). As an additional physiological indicator of WS, the transpiration rate (*E*) was also measured (Fig. 1). Under WW conditions, *E*-values were not statistically different and equal to 2.7±0.3 and 2.4±0.2 mmol H_2_O m^–2^ s^–1^ in M4 and 101.14 genotypes, respectively, and remained relatively constant throughout the treatment period. On the other hand, upon application of the WS treatment, which led to a reduction in the proportion of field capacity to ~30% at 6 DASI, 101.14 plants dropped to extremely low *E*-values, whereas M4 plants maintained values of ~20% with respect to the control (Fig. 1a). In order to ascertain whether typical signals induced by drought were already present at the early phases of stress and, therefore, if the rootstock genotypes behaved similarly in terms of the perception of water deprivation, the leaf ABA content was analysed throughout the treatment period. (Fig. 1b). In the leaves of water-stressed 101.14 plants, the ABA content was significantly higher than in the control plants (WW) at T1 (fold change=1.58), whereas in water-stressed M4 plants no differences in ABA content were noted until T2. At T2, both genotypes accumulated a conspicuous amount of ABA, although 101.14 reached higher values compared with M4. The ABA level remained almost constant in water-stressed 101.14 plants between T2 and T4, while it continued to increase in water-stressed M4 plants.

### PN400024 is a suitable reference for mapping both the 101.14 and M4 transcriptome

The interspecific hybrids M4 and 101.14 were obtained by crossing selected North American wild grapevine species (*V. riparia*, *V. rupestris*, and *V. berlandieri*) with the European cultivated species (*V. vinifera*). This raised the question of whether potential differences between the sequence of predicted genes in the PN40024 reference genome (Jaillon *et al.*, 2007) and orthologous sequences in the 101.14 and M4 genomes may impair the mapping of mRNA reads to the reference genome and compromise the robustness of the data. To address this issue, the resequencing of the M4 and 101.14 genomes was performed. On average, only one variant every 200 bases was found both in M4 and in 101.14, suggesting that the PN40024 genome should be a suitable reference for read mapping (Supplementary Results S1 at *JXB* online). The genome reference was corrected according to the SNPs and small indels (In/Dels) identified in both resequenced genomes, and the resulting sequences are available at http://genomes.cribi.unipd.it/grape/serres/. Furthermore, when the mRNA-Seq reads were mapped on their corresponding corrected genomes, only a negligible increase of alignments (~1%) was obtained as compared with the mapping onto PN40024. Based on these results, all RNA-Seq reads were mapped on the PN40024 reference genome, thus making the comparison of the different samples more manageable. A further question was whether the reference PN40024 genes are present in both the genotypes. To investigate this point, the genomic reads of M4 and 101.14 were mapped independently on the PN40024 reference genome, allowing multiple mapping, with the same stringency used for RNA-Seq mapping. The results indicated that all the genes of the reference genome were covered both by the M4 and by the 101.14 reads; the only exception being a single gene of unknown function (VIT_00s1914g00010) that seemed to be absent in both the 101.14 and M4 genotypes.

In order to obtain a snapshot of changes in the transcriptome of the two rootstock genotypes over the entire stress period, an mRNA-Seq analysis was performed on all 72 samples as described in the Materials and methods. This produced ~4.8 billion paired-end reads (75 and 35 nucleotides in length for forward and reverse reads, respectively), with the total number of reads produced for each time point ranging from 29 to 82 million paired-end reads and a median of 45 million reads (Supplementary Table S1 at *JXB* online). On average, 90% of the reads passed the quality control test (filtered based on read length after trimming the low quality bases) and were mapped to the PN40024 12X v1 grape reference genome (http://genomes.cribi.unipd.it/grape/), producing between 10 and 37 million unique mapping reads depending on the sample (Supplementary Tables S1, S2).

### Drought is the main factor driving differential gene expression in roots but not in leaves

**Fig. 2.**
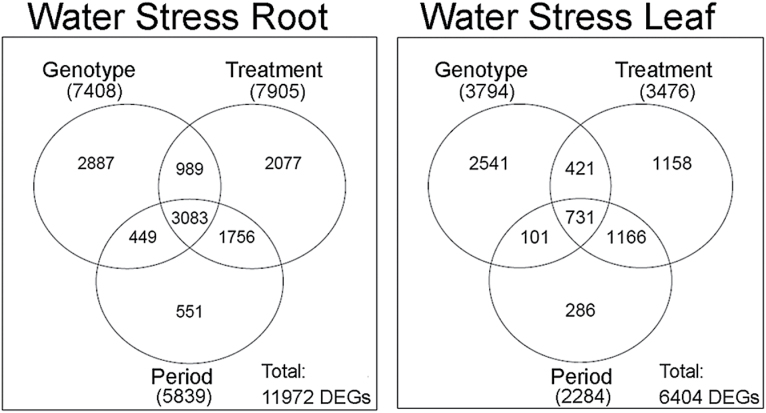
Venn diagrams of DEGs resulting from multifactorial analyses conducted on root and leaf tissues under WS treatment, according to a *P*<0.05. The number of DEGs influenced by each component is given in parentheses; the total number of genes influenced by root and leaf tissues is also indicated (‘Total’).

An important step in the statistical analysis aimed at identifying DEGs upon WS in both genotypes was estimating the influence of different independent components such as the genotype (G), the treatment (T), and the time point considered (P) on the transcriptomic profile. Thus, a multifactor analysis was conducted on mRNA-Seq data sets obtained from WS and WW root and leaf tissues, in order to evaluate both the singular (G, T, P) and combined (G:T, G:P, T:P, G:T:P) impact of each component on DEGs according to an FDR-adjusted *P*-value <0.05 (Supplementary Table S3 at *JXB* online). The Venn diagram shown in Fig. 2 summarizes the impact of each component, indicating the number of genes specifically influenced by a single variable and those influenced by more than one variable. In root tissues undergoing WS, the total number of DEGs influenced by each single G, T, and P component was 7408, 7905, and 5839, respectively (Fig. 2). In leaf, there were 3794 DEGs for G, 3476 for T, and 2284 for the P component (Fig. 2). In other words, considering for example WS roots (Fig. 2), 2887 genes were differentially expressed only because of the effect of genotype, regardless of the effect of treatment (WW or WS) or the time of sampling (T1–T4) considered. Conversely, 2077 genes were differentially expressed in response to application of the WS treatment, independent of the genotype (101.14 or M4) and of the sampling time (T1–T4). Finally, 551 genes appeared to be developmentally regulated and show differential expression over the period of the experimental treatment independent of the genotype or the treatment applied.

The multifactorial analysis highlighted a strong influence of the organ (root or leaf) on the final number of DEGs. Thus, water-stressed roots showed approximately twice the number of DEGs as water-stressed leaves (Fig. 2). Interestingly, the treatment component had the major influence on gene expression in roots, showing the highest number of DEGs (7905) compared with all other components, whereas the genotype component showed the highest impact on leaves (3794 DEGs).

**Fig. 3.**
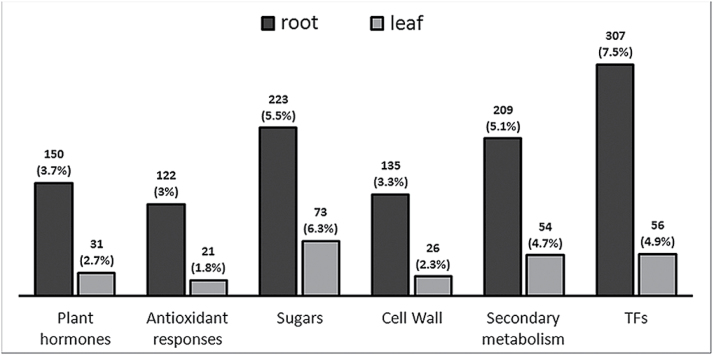
Analysis of ontology categories of genes differentially expressed in M4 and 101.14 roots and leaves under drought conditions related to plant hormone-, antioxidant response-, sugar-, cell wall-, secondary metabolism-, and transcription factor-related GO terms. The number and percentage of DEGs (resulting from multifactorial analysis) belonging to each category are provided.

In order to identify specific metabolic pathways differentially affected by drought stress in M4 and 101.14 roots and leaves, those DEGs captured by multifactorial analysis common to either the G and T components or common to the G+T+P components were associated with their respective GO terms. This represented 4072 and 1152 genes in roots and leaves, respectively. GO terms were grouped into macro-categories (Fig. 3), and the complete lists of GO terms of DEGs identified in roots and leaves are reported in Supplementary Tables S4 and S5 at *JXB* online, respectively. Amongst root-related macro-categories, ‘transcription factors’ accounted for the highest number of DEGs (307 genes out of 4072 DEGs, corresponding to 7.5%), followed by 223 and 209 DEGs related to ‘sugars’ (5.5%) and ‘secondary metabolism (5.1%), respectively. The GO categories ‘plant hormones’ (150 DEGs, 3.7%), ‘cell wall’ (135 DEGs, 3.3%), and ‘antioxidant responses’ (122 DEGs, 3%) accounted for fewer DEGs under WS conditions.

DEGs identified in water-stressed leaves were represented in different GO macro-categories compared with roots. Transcripts related to ‘sugars’ were the most represented, with 73 DEGs (6.3%), whereas ‘transcription factors’ and ‘secondary metabolism’ categories accounted for 56 (4.9%) and 54 (4.7%) of the DEGs, respectively. As observed in roots, ‘plant hormones’ (31 DEGs, 2.8%), ‘antioxidant responses’ (21 DEGs, 2%), and ‘cell wall’ (26 DEGs, 2.3%) categories were less represented.

### Drought differentially affects stilbene and flavonoid metabolism in M4 and 101.14 plants

In order to better capture differential expression patterns between 101.14 and M4 genotypes, and to characterize the conservation or divergence of co-expression between genes with a related function, a recently developed approach, termed DCA, was implemented (Ihmels *et al.*, 2005; Lelandais *et al.*, 2008; Cohen *et al.* 2010). This approach allows the identification of co-expression clusters within a given gene set and to assign each of these clusters to one of four correlation categories defined on the basis of the level of conservation between two genotypes: ‘full’, ‘partial’, ‘split’, or ‘absent’.

**Fig. 4.**
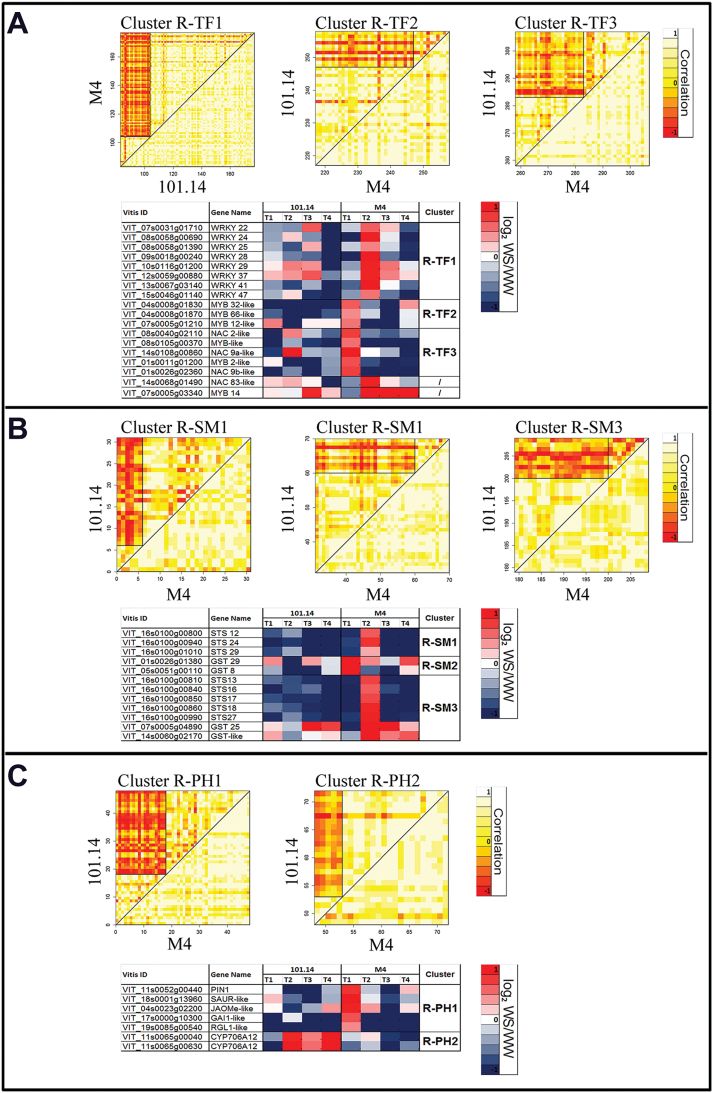
Differential cluster analysis (DCA) conducted on transcription factor (A; R–TF), secondary metabolism (B; R–SM), and plant hormone (C; R–PH) GO categories enriched in root DEGs. Correlation is graphically represented in the heat map. White boxes indicate a complete correlation amongst transcripts (value=1), yellow boxes indicate no correlation (value=0), and red boxes indicate anti-correlation (value= –1). DEGs showing different behaviour between the two genotypes are listed in tables and associated with a graphical representation of their expression (log_2_ WS/WW) within the whole stress kinetic. Blue boxes represent down-regulation, whereas red boxes represent up-regulation.

**Fig. 5.**
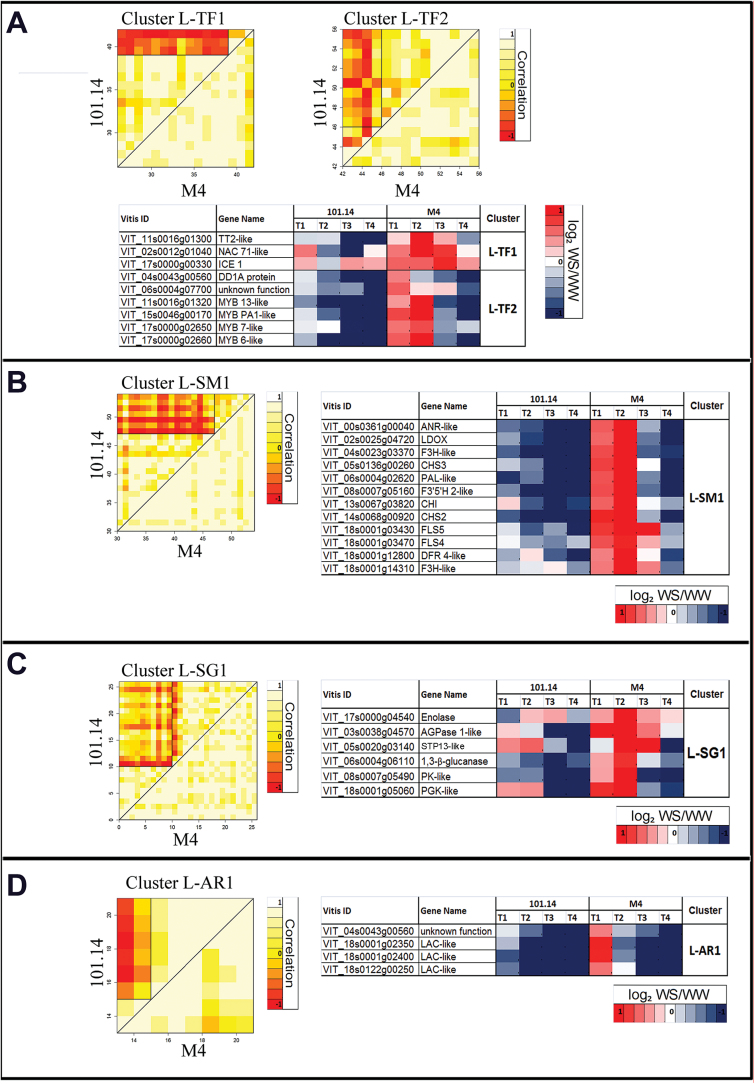
Differential cluster analysis (DCA) conducted on sugar (A; L–TF), secondary metabolism (B; L–SM), transcription factor (C; L–SG), and antioxidant response (D; L–AR) GO categories in leaf DEGs. Correlation is graphically represented in the heat map. White boxes indicate a complete correlation amongst transcripts (value=1), yellow boxes indicate no correlation (value=0), and red boxes indicate anti-correlation (value= –1). DEGs showing different behaviour between the two genotypes are listed in tables and associated with a graphical representation of their expression (log_2_ WS/WW) within the whole stress kinetic. Blue boxes represent down-regulation, whereas red boxes represent up-regulation.

Basically, the DCA method allows both similarities and differences in the ﬁne structure of co-regulation patterns between two genotypes to be systematically characterized and assigned to one out of four conservation categories: full, partial, split, or no conservation of co-expression. In a ‘full’ conservation class, a cluster of genes, which are correlated together in one of two genotypes, also correlate in the other. In a ‘partial’ conservation class, only a subset of those genes which appear to correlate in one genotype also correlate in the other. A ‘split’ conservation class describes a condition in which genes which are grouped in a single correlation cluster in one genotype, are divided into two (or more) correlation subclusters in the other. Finally in case of ‘absent’ or ‘no correlation’, only one genotype shows correlation between a given group of genes whereas the other does not (for further details, see Supplementary Method S3 at *JXB* online). For this approach only those G–T and G–T–P common DEGs sharing membership of the same GO categories as previously identified were considered and they were associated with their expression values provided as log_2_ WS/WW tissues (Supplementary Table S6). Clusters showing the most interesting features in both root and leaf tissues are reported in Figs 4 and 5. Again, the data confirmed that different organs display different responses to stress at the gene expression level. In fact, while changes in expression related to ‘transcription factors’ and ‘secondary metabolism’ ontologies were common features both for roots (Fig. 4a, b) and for leaves (Fig. 5a, b), the ‘plant hormones’ category (Fig. 4c) was significantly modulated only in roots, whereas the ‘sugars’ category (Fig. 5c) was affected exclusively in leaves.

Within the transcription factors (TFs) category (Fig. 4a), DEGs in roots were subdivided into three clusters that highlight different expression kinetics: root (R)–TF1 (reference 101.14), R–TF2, and R–TF3 (reference M4) (Supplementary Methods S3 at *JXB* online). Genes belonging to these categories were uniformly co-expressed in the reference genotype. In contrast, in the target genotype, they were split into two distinct secondary clusters, one of which was similar in both 101.14 and M4, with the other one showing different behaviour for several genes, indicating a ‘partial’ conservation. Genes of particular interest within these clusters were those belonging to the *WRKY* (R–TF1, ‘split’), *MYB* (R–TF2, ‘split’), and *NAC* (R–TF3, ‘partial’) families. *WRKY* TFs showed a different behaviour between the two genotypes, being strongly up-regulated at T2 only in M4 (Fig. 4a). Five MYBs and three NAC TFs were induced at T1 in M4 under WS, but were significantly down-regulated in 101.14 (Fig. 4a). In leaves, amongst TFs [leaf (L)–TF], MYBs were the most represented gene family showing opposite expression kinetics in M4 and 101.14 genotypes. In the L–TF2 cluster (Fig. 5a), four MYB TFs were induced at T1 and T2 only in M4 upon WS. In contrast, these TFs showed a lower expression in 101.14 WS leaves than in WW leaves (Fig. 5a).

**Fig. 6.**
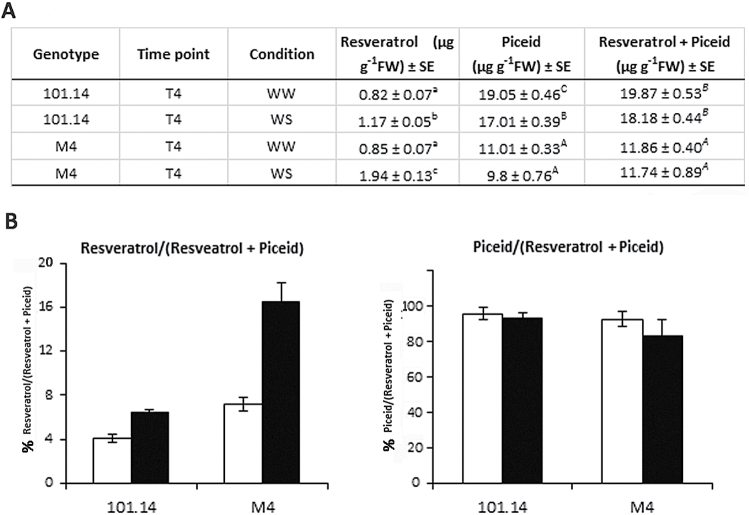
(A) Quantification of *trans*-resveratrol and *trans*-piceid in 101.14 and M4 roots at T4 in well-watered (WW) and water stress (WS) conditions. (B) Percentage of resveratrol and piceid fraction with respect to their sum in WW (withe bars) and WS (black bars) conditions. Values are calculated as the mean (*n*=4). Vertical bars indicate the standard error (SE). Letters indicate statistical differences in (two-tailed unpaired) Student’s *t*-test at *P*<0.01.

Another GO category giving interesting results in the DCA analysis was ‘secondary metabolism’ (SM) (Figs 4b, 5b). In roots, most of the transcripts belonging to this ontology encoded glutathione *S*-transferases (GSTs) and stilbene synthases (STSs) (clusters R–SM1, 2, and 3). Based on the nomenclature proposed by Vannozzi *et al.* (2012), eight *VvSTS* transcripts (*VvSTS12*, *13*, *16*, *17*, *18*, *24*, *27*, and *29*) were found to be significantly up-regulated in M4 stressed roots at T2, whereas they were generally down-regulated in 101.14 (Fig. 4b) for both R–SM1 (no conservation) and R–SM3 (partial conservation) clusters. Based on the differences observed in *VvSTS* transcript abundance, the concentration of free and conjugated forms of stilbenes was measured in the roots of the two genotypes. As a general observation, looking at the sum of resveratrol and piceid concentration, the results indicated that 101.14 roots accumulate more stilbenes than M4 in both control and stress conditions. Moreover, water stress did not appear to affect the sum of resveratrol and piceid significantly in either genotype (Fig. 6a). Only a slight decrease was registered, mainly due to a decline in piceid content. Looking more in detail and considering singularly both the fraction of resveratrol and piceid with respect to their sum, it must be noted that the piceid fraction remained almost unaltered in both genotypes, whereas the resveratrol fraction showed a marked increase upon WS. This was particularly evident in the M4 genotype (Fig. 6b) where the percentage of resveratrol on the sum of resveratrol and piceid passed from 6% to 16%.

The observation that *VvSTSs* genes appear to be co-expressed with a number of *WRKY* TFs (Fig. 4a) raised the question of whether these TFs might be involved in the transcriptional regulation of *VvSTS* genes. An *in silico* search for putative *cis*-elements in the promoter regions of *VvSTS* genes that were identified to be up-regulated at T2 further supported this hypothesis. Random motifs of 4–7 mers were retrieved from 10 000 independent promoter sequences of PN40024, M4, and 101.14 genotypes. Amongst these, only those *cis*-elements classified as W-BOX were considered for statistical analysis (Supplementary Fig. S2 at *JXB* online). A *t*-test was done comparing the frequencies of W-BOXs in the promoter of *VvSTS* genes and in another 10 000 randomly chosen promoters in each genotype. A significant difference (*P*<0.05) was only found in M4, where almost all *VvSTS* promoters (with the exception of *VvSTS12*, VIT_16s0100g00800) exhibited a higher number of W-BOX elements compared with 101.14. The number of W-BOX elements was significantly correlated to the expression levels of *VvSTS* genes (*R* ^2^=0.76), with members characterized by a higher number of W-BOX elements in the promoter also showing a higher expression (Supplementary Fig. S2).

Regarding the L–SM clusters, these included 12 genes related to the flavonoid metabolic pathway, all induced at T1 and strongly expressed at T2 in M4 water-stressed leaves (Fig. 5b). Amongst these were: (i) phenylalanine-ammonia-lyase (*PAL*), chalcone synthase (*CHS*), and chalcone isomerase (*CHI*) genes, which catalyse the early steps of the general phenylpropanoid pathway; (ii) flavonoid 3-hydroxylase-related genes (*F3’5’H 2-like* and two *F3H-like*); (iii) *flavonol synthase 4* (*FLS4*) and *FLS5*, which lead to flavonol biosynthesis; and (iv) dihydroflavonol 4-reductase (*DFR 4-like*), leucoanthocyanidin dioxygenase (*LDOX*), and anthocyanidin reductase (*ANR*), which lead to anthocyanin biosynthesis.

The ‘plant hormones’ (PH) category also showed an interesting pattern of expression in roots (Fig. 4c). Amongst expression clusters identified through the DCA analysis within this GO category, one cluster, namely R–PH1, belonged to the ‘no conservation’ class whereas the R–PH2 primary cluster displays a ‘split conservation’. Cluster R–PH1 is composed exclusively of transcripts up-regulated in M4 at T1 and involved in auxin transport (*PIN1*), auxin signal transduction (*SAUR-like*), JA biosynthesis (*JAOMe-like*), and GA signal transduction (*GAI1* and, *RGL1*). In contrast, R–PH2 contained genes induced exclusively in 101.14 at later time points (T2, T3, and T4) and included two ABA 8’-hydroxylase-related genes, encoding enzymes involved in ABA catabolism. It is worth noting that the opposite behaviour of these genes in the two rootstock genotypes (induced by WS in 101.14 and repressed in M4) was not observed for genes involved in ABA biosynthesis and signalling (Supplementary Fig. S3 at *JXB* online).

The ‘sugars’ category (L–SG) was found to be the most highly represented in terms of DEGs in leaf tissue (Fig. 3). Six transcripts belonging to the L–SG1 cluster (Fig. 5c) showed an induction in water-stressed M4 plants, although with different patterns of expression. In contrast, these genes were all down-regulated in water-stressed 101.14 plants across the whole stress treatment. The leaf ‘antioxidant responses’ (L–AR) category showed split conservation between 101.14 and M4 genotypes, with three genes belonging to the laccase (*LAC*) family displaying an induction limited to M4 leaves at the early stage of WS (T1) (Fig. 5d).

## Discussion

The study provides a genome-wide description of the transcriptional response induced by WS in two grapevine rootstock genotypes, M4 and 101.14, which display contrasting tolerance to drought. This study not only compares the transcriptomic responses of drought-susceptible and drought-tolerant genotypes, but also provides a comparative characterization of the WS responses of the underground (roots) and aerial (leaves) parts of the plant. To replicate as accurately as possible changing conditions normally found in the field and thus capture the adaptive response to drought, WS was progressively imposed on the plants by gradually reducing water availability to reduce field capacity from 80% to 30% over a 10 d period (Supplementary Fig. S1 at *JXB* online). In this regard, the experimental design employed here is markedly different from that used in previous studies (Cramer *et al.*, 2007), where water supply was completely halted from the beginning of the stress experiment.

In both 101.14 and M4 WS plants, the reduction of *E* was accompanied by a significant increase in leaf ABA. However, although ABA levels at severe stress (T4) were almost similar in the two rootstock genotypes, the pattern of accumulation observed was different, with 101.14 starting to increase the concentration of hormone from the first stages of water deprivation (T1), reaching a plateau already at T2. The fast and high accumulation of ABA is in accord with the most marked decline of stomatal conductance observed, under the same experimental conditions, in 101.14 in comparison with M4 by Meggio *et al.* (2014). All these pieces of evidence (in the present study and in Meggio *et al.*, 2014) suggest that 101.14 adopts a more conservative strategy to cope with WS compared with M4, which maintains a higher transpiration rate (Fig. 1) also upon severe WS.

To obtain a more comprehensive understanding of physiological responses to drought a whole-transcriptome analysis was performed in both water-stressed and unstressed plants. Taking into account that M4 and 101.14 are interspecific hybrids, as a first step their genomes were resequenced to verify if PN40024 was a suitable reference for mapping the mRNA-Seq reads from the hybrids. Resequencing revealed that (i) both the genotypes have a low and comparable frequency of SNPs (Supplementary Results S1 at *JXB* online) as already observed by Myles *et al.* (2010) amongst different *Vitis* species and (ii) compared with PN40024 gene annotation, genotype gene content is quite similar. Taken together, these findings indicated that PN40024 is a suitable reference for performing gene expression analysis. However, it is also worth noting that the technology used in this study does not allow exclusion of gene families that have been expanded or condensed in the different genotypes.

Multifactorial analyses (Fig. 2) on whole leaf and root transcriptome data sets enabled better definition of the specific influence of the genotype and stress treatment on the transcriptome, and allowed those DEGs whose expression is only linked to the contribution of a single component to be filtered out. In other words, it was possible to exclude those genes whose differential expression was caused just by the ‘genotype’ or ‘treatment’ and consider those affected by the contribution of both variables (McCarthy *et al.*, 2012).

The comparison of leaf and root expression data obtained by multifactorial analyses (Fig. 2; Supplementary Table S3 at *JXB* online) indicated that in roots (Fig. 2) the ‘treatment’ factor is the main variable explaining differential gene expression, whereas in leaves (Fig. 2) the ‘genotype’ appears to be the predominant factor. This observation is not surprising, given that the root system is the first organ to perceive water deprivation and actively respond to this stress (Frensch et al., 1997), and thus the type of treatment represents the main variable influencing gene expression in this organ. The opposite is true in the aerial part of the plant, where the ‘genotype’ factor appears to have a major effect when compared with the other components. A possible explanation for this observation relies on the fact that, if roots mediate the early perception of the stress, the secondary signals, which are produced by these organs and are transmitted to leaves, are strictly related to the modality by which the apical part of the plant responds to these perturbations and this is mainly conditioned by the genotype. The role of roots in the initial perception of WS is indicated by the high expression of genes involved in ABA biosynthesis and signalling in both genotypes compared with leaves (Supplementary Fig. S3). Similar results were also reported in Cabernet Sauvignon vines grafted on Ramsey, where the expression of two main genes associated with ABA synthesis, *NCED1* and *NCED2*, was found to be more pronounced in roots compared with leaves and to be inversely correlated to water supply (Speirs *et al.*, 2013). Since ABA has been claimed to play a major role in root to shoot signalling upon WS (Dood, 2005), it is possible that the increase in its concentration observed in both 101.14 and M4 leaves (Fig. 1b) is mainly due to its transportation from roots rather than its biosynthesis at the leaf site, as already reported by Speirs *et al.* (2013). In this context, the observation that ABA content in 101.14 leaves reached a plateau at T2–T4 whereas it continued to increase in M4 leaves (Fig. 1b), although ABA biosynthetic genes were similarly induced in both rootstock genotypes (Supplementary Fig. S3), could be a possible consequence of the different behaviour observed in ABA catabolism at the root level. This may indicate the existence of a different control of ABA homeostasis within the two genotypes under study. In fact the results indicated that two orthologues of the *Arabidopsis* CYP706 and CYP707 genes, which encode the major enzyme involved in ABA catabolism during dehydration (Speirs *et al.*, 2013; Liu *et al.*, 2014), were induced in response to water-stressed 101.14 roots at all time points following T2, whereas they were maintained constantly down-regulated in M4 compared with the control (Fig. 4; Supplementary Fig. S3). Differences in the behaviour of genes involved in hormone homeostasis observed between the two rootstocks were not limited to genes related to ABA, but also included other hormones. For example, WS induced the expression of JA- and GA-related genes only in M4 water-stressed roots (Fig. 4c), whereas these transcripts were down-regulated in stressed roots of 101.14. Methyljasmonate (MeJA) mediates many developmental processes and defence responses to biotic and abiotic stresses in plants (Ismail *et al.*, 2012). A positive correlation between drought and MeJA biosynthesis was also observed in rice and chickpea, stressing the putative role of this hormone in WS-induced responses (Kim *et al.*, 2009; De Domenico *et al.*, 2012).

DCA analysis on genes belonging to the ‘sugar’ category highlighted the existence of a gene orthologous to an *Arabidopsis* sugar transporter protein (*AtSTP13/MSS1*) which showed a higher induction in M4 stressed leaves compared with 101.14. Although the role of *AtSTP13* has not been fully characterized, this gene seems to be drought responsive and, similarly to many other sucrose transporters, it codes for a Suc/H^+^ symporter which could be potentially involved in the phloem loading and in the long-distance transport of soluble sugars from source organs to sinks such as roots (Kühn and Grof, 2010). This hypothesis is congruent with the observation that M4 WS roots accumulate a higher level of soluble sugars with respect to 101.14 (Meggio *et al.* 2014).

In both roots and leaves, a significant number of DEGs belonged to the ‘secondary metabolism’ category, although being involved in different metabolic pathways. In leaves, M4 stressed plants showed an induction of many structural genes involved in the flavonoid pathway (Fig. 5b). Conversely, in roots, plants accumulated both transcripts (Fig. 4b) and proteins (Luca Espen, personal communication) corresponding to stilbene synthases (VvSTS), responsible for the biosynthesis of the 3-hydroxy-*trans*-stilbene, better known as resveratrol. Although in 101.14 the sum of resveratrol and its glycosylated form (piceid) reached values always higher than in M4 both in WW and in WS conditions, WS significantly affected the balance between resveratrol in its free and glycosylated form, particularly in M4 (Fig. 6b). This observations seems to suggest that upon WS, M4 stilbene metabolism is addressed preferentially towards the accumulation of resveratrol in its free form. Based on previous studies, *trans*-resveratrol appears to have a higher impact in scavenging the oxidative stress related to various stresses with respect to other compounds including its glycosides (Waffo-Teguo *et al.*, 1998).

The observed co-expression between a number of *VvWRKY* transcription factors (*VvWRKY24/28/29/37/41*) and *VvSTS* genes in M4 (Fig. 4a, b) raises the question of a possible role for WRKYs in the regulation of *VvSTS* gene expression in *Vitis* species (Wang *et al.*, 2014). This hypothesis is further supported by the presence of W-BOX *cis*-regulatory elements in the promoter region of *VvSTS* genes in M4 and 101.14 (Supplementary Fig. S3 at *JXB* online) and by the fact that the higher expression of *VvSTS* genes under WS in M4 correlates well with the significantly higher frequency of W-BOX elements in their promoter regions compared with 101.14 and PN40024 (Supplementary Fig. S2). A recent study by Gao *et al.* (2013) also demonstrated a strict association between the expression level of certain genes and the *cis*-regulatory number of domains within their promoter sequences.

In the aerial part of M4 WS plants, the up-regulation of genes involved in flavonoid biosynthesis, such as *CHS2*, *CHS3*, *F3H*, *FLS1*, and *LDOX*, was paralleled by an induction of specific R2R3-MYB TFs (Fig. 5a, b). Although a relationship between R2R3-MYB TFs and flavonoid biosynthetic genes is well documented (Czemmel *et al.*, 2012; Ambawat *et al.*, 2013), it was not possible to detect any difference in MYB-related *cis*-element content within the promoter sequences of these genes in the two genotypes (data not shown).

Stilbenes and flavonoids have ROS-scavenging activity that protects against oxidative damage and controls ROS levels, which is mandatory for plant survival in the presence of abiotic stresses (Brunetti *et al.*, 2013; Höll *et al.*, 2013). Stilbenoids (resveratrol in particular) are powerful defence antioxidant molecules found in several species, and their accumulation is particularly high in grapevine (Jeandet et al., 2010, 2013; Vannozzi *et al.*, 2012; Höll *et al.*, 2013; Stuart and Robb, 2013). It has been suggested that flavonoids, whose biosynthetic genes are induced in M4 leaves under WS, act as antioxidants in plant response to oxidative stresses (Ramakrishna and Ravishankar, 2011; Brunetti *et al.*, 2013). Flavonoids may also reduce the activity of ‘primary’ ROS scavenger enzymes (i.e. superoxide dismutase and catalase) in the chloroplast (Mullineaux and Karpinski, 2002; Brunetti *et al.*, 2013). In addition, flavonoids are capable of quenching H_2_O_2_ and other free radicals, thus protecting the chloroplast membrane from oxidative damage by stabilizing membranes containing non-bilayer lipids (Agati *et al.*, 2012).

The present data suggest that in addition to the activation of ‘primary mechanisms’ of ROS scavenging, the drought-tolerant M4 rootstock genotype may also induce ‘secondary mechanisms’ leading to the biosynthesis of other types of secondary compounds in roots and leaves.

**Fig. 7.**
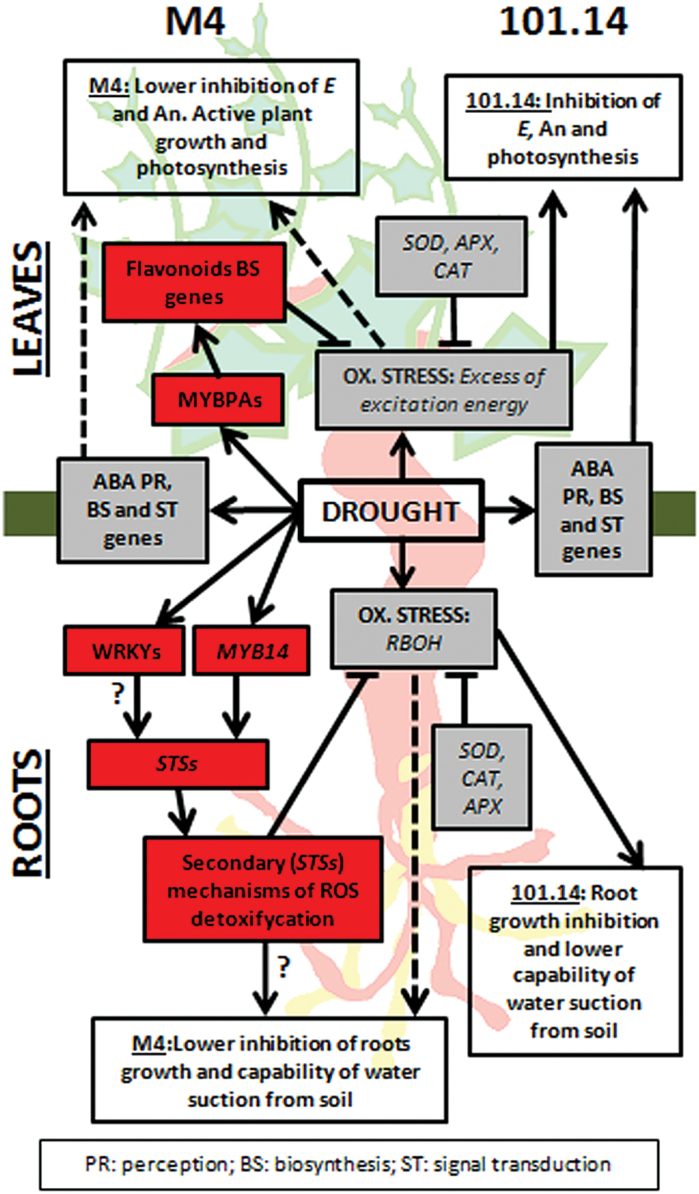
Hypothetical model summarizing the events occurring in leaves and roots of M4 and 101.14 upon WS. Grey and red boxes list molecular responses to WS that are common to both genotypes and M4 specific, respectively. White boxes report physiological events associated with WS occurring in root (lateral roots growth and water suction from soil) and leaves (transpiration, *E*; net-assimilation, *A*_n_; photosynthesis) of both genotypes. Dashed lines indicate the lower impact of WS on the physiological events in roots and leaves observed in M4.

In conclusion, this study provides a comprehensive description of the transcriptomic responses to drought in roots and leaves of two grapevine genotypes with different tolerances to WS. In contrast to previous studies (Cramer *et al.*, 2007; Tattersall *et al.*, 2007; Tillett *et al.*, 2011), responses to WS that are common to susceptible and tolerant plants were not considered, but rather the focus was on genes whose expression is strictly related to the tolerant genotype. On this basis, it is proposed that the drought tolerance displayed by the M4 genotype could be associated with an enhanced capacity to scavenge ROS produced under stress conditions and that this may be mainly conferred by structural variations in the promoter of genes involved in stilbene biosynthesis (Fig. 7). In water-stressed M4 plants, the higher ROS detoxification ability could allow lateral root growth to be maintained, resulting in higher water uptake capacity from the soil, as previously observed by Tsukagoshi (2012). Likewise, at the leaf level, a higher *E* in the drought-tolerant genotype would promote active plant growth and photosynthesis. In contrast, in 101.14, where the oxidative stress is not efficiently counteracted, the functionality of roots and leaves is strongly impaired (Fig. 7).

The candidate genes identified in this study to be putative factors underlying the better adaptation of the M4 genotype to WS will be further validated using an association genetics approach. The expression of selected candidate genes will be evaluated on a large range of genotypes exhibiting differential responses to WS in order to assess whether the drought tolerance strategies operating in M4 are conserved in other genotypes and, if so, to use the identified genes as functional markers (Poczai *et al.*, 2013) for the selection of WS-tolerant grapevine rootstocks.

## Supplementary data

Supplementary data are available at *JXB* online.

Figure S1. Schematic representation of the experimental plan.

Figure S2. W-BOX *cis*-elements in six *VvSTS* promoters.

Figure S3. Heat maps showing ABA-related genes in roots and leaves of M4 and 101.14 genotypes.

Table S1. Summary of mRNA-Seq read number.

Table S2. Summary of read number after pairing (F3+F5).

Table S3. List of DEGs identified by multifactor analysis in root and leaf tissues.

Table S4. GO terms associated with DEGs identified by multifactor analysis in roots.

Table S5. GO terms associated with DEGs identified by multifactor analysis in leaves.

Table S6. Pairwise comparisons of leaf and root tissues of both 101.14 and M4 rootstocks upon water stress

Supplementary Results S1. M4 and 101.14 genome resequencing.

Supplementary Methods S1. mRNA sample preparation and sequencing.

Supplementary Methods S2. ABA and stilbene quantification.

Supplementary Methods S3. Differential cluster analysis (DCA).

Supplementary Data
