# Supplementary material for: Comprehensive transcript profiling of two grapevine rootstock genotypes contrasting in drought susceptibility links the phenylpropanoid pathway to enhanced tolerance
Source: J Exp Bot. 2015 Jun 2;66(19):5739–52. doi: 10.1093/jxb/erv274 (PMC4566973; doi:10.1093/jxb/erv274)
Supplement: Supplementary Data [file supp_66_19_5739__index.html]

Comprehensive transcript profiling of two grapevine rootstock genotypes contrasting in drought susceptibility links the phenylpropanoid pathway to enhanced tolerance — Comprehensive transcript profiling of two grapevine rootstock genotypes contrasting in drought susceptibility links the phenylpropanoid pathway to enhanced tolerance — Supplementary Data 

# Comprehensive transcript profiling of two grapevine rootstock genotypes contrasting in drought susceptibility links the phenylpropanoid pathway to enhanced tolerance

## Supplementary Data

Data files

- Supplementary Data - Supplementary Data
- Supplementary Data - Supplementary Data
