# Supplementary material for: Comprehensive transcript profiling of two grapevine rootstock genotypes contrasting in drought susceptibility links the phenylpropanoid pathway to enhanced tolerance
Source: J Exp Bot. 2015 Jun 2;66(19):5739–52. doi: 10.1093/jxb/erv274 (PMC4566973; doi:10.1093/jxb/erv274)
Supplement: Supplementary Data [file supp_erv274_jexbot145623_file001.pdf]

***Journal of Experimental* Botany Supplementary Data**

Article title: Comprehensive transcript profiling of two grapevine rootstock genotypes contrasting in drought susceptibility links phenylpropanoid pathway to enhanced tolerance

Authors: Massimiliano Corso, Alessandro Vannozzi, Elie Maza, Nicola Vitulo, Franco Meggio, Andrea Pitacco, Andrea Telatin, Michela D'Angelo, Erika Feltrin, Alfredo Simone Negri, Bhakti Prinsi, Giorgio Valle, Angelo Ramina, Mondher Bouzayen, Claudio Bonghi and Margherita Lucchin.

The following Supplementary Data are available for this article:

Supplementary Fig. S1 Schematic representation of the experimental plan. The WS was gradually imposed by decreasing the water-availability in pots from 80% to 30% of field capacity, whereas WW plants, used as control, were maintained to 80% of field capacity. In order to attenuate the fluctuation in soil water content, pots were weighed and then the quantity of water adequate to reach/ maintain the desired fraction of soil field capacity was added. The procedure was repeated twice a day, at 8:00 am and at 6:00 pm.

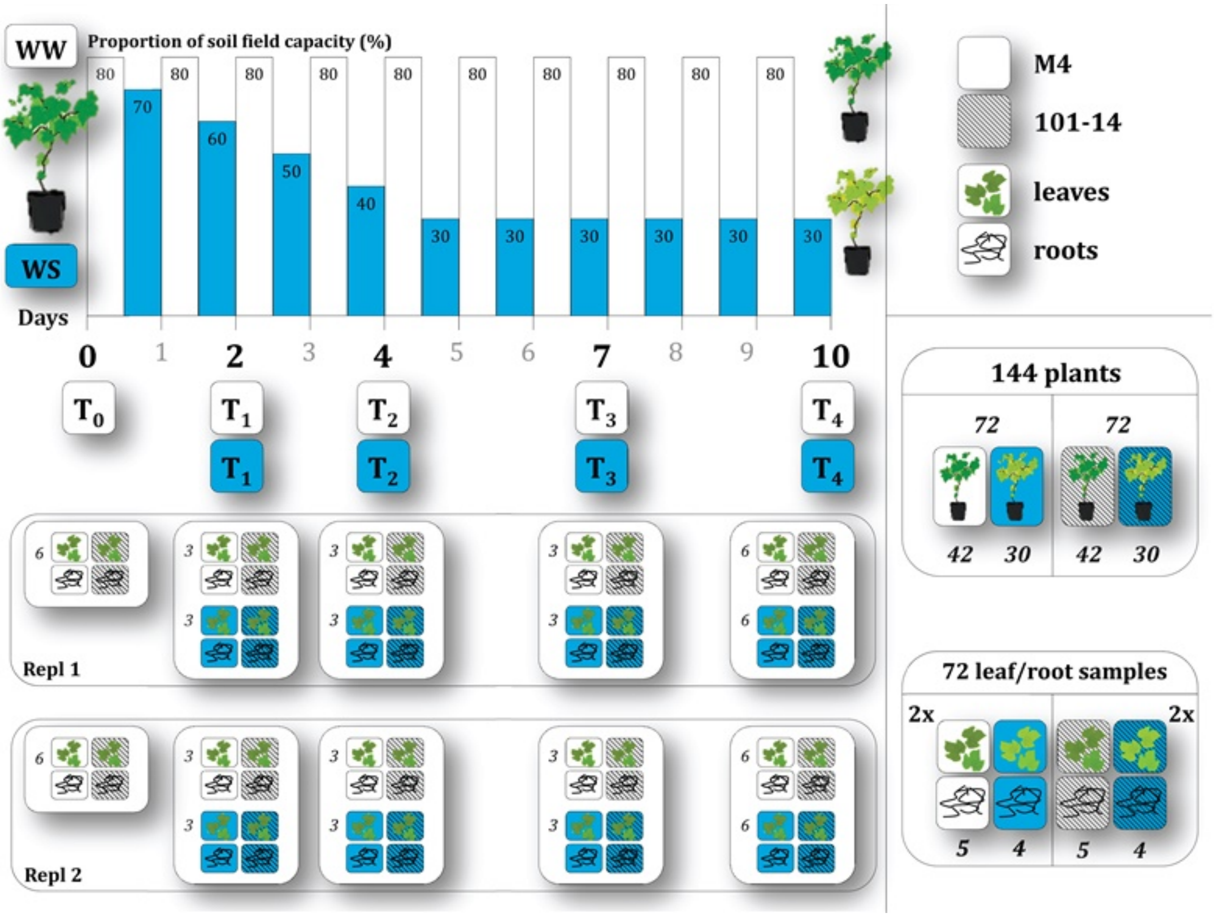

WW
Proportion of soil field capacity (%)
80
70
60
50
40
30
WS
Days
0 1 2 3 4 5 6 7 8 9 10
T_0
T_1
T_2
T_3
T_4
M4
101-14
leaves
roots
144 plants
72
72
42
30
42
30
72 leaf/root samples
2x
2x
5
4
5
4
Repl 1
Repl 2

Supplementary Fig. S2 W-BOX cis-elements in six *VvSTS* promoters.

Figure S2. WBOX cis-elements in six *VvSTSs* promoters. (a) Frequencies of W-BOXs motif detected in VvSTS DE genes and in 10000 promoters (2Kb sequences) randomly selected from PN40024, 101.14 and M4 genotypes. A t-test was carried out comparing the frequencies of WBOXs in STSs and in 10000 random promoters separately for each genotype. (b) Number of each W-BOX cis-element in M4 and 101.14. C. Relative expression of VvSTS12/16/17/18/27/29 genes (blue bars). Histogram represent the log2M4/101.14 only for WS plants at T2 (4 DASI). Difference between number of total W-BOX in M4 and 101.14 (nrM4 - nr101.14) are given in brackets. D. Correlation between log2M4/101.14 (WS plants) and number of WBOX M4 - 101.14.

(a)

WBOX	Cis-element frequency				
	10000 random promoters			STSs DE genes	
	PN40024	101.14	M4	101.14	M4
TGAC	5.3	5.3	5.3	10.3	12.7
TTGAC	1.9	1.9	2.0	2.8	6.3
TTTGAC	0.8	0.8	0.8	3.3	3.8
TGACC	1.1	1.1	1.1	4.8	8.7
TGACT	1.7	1.7	1.7	3.5	7.5
TTTGACC/T	0.2	0.2	0.2	3.0	5.3
TTGACC	0.4	0.4	0.4	0.5	1.2
TTGACT	0.7	0.7	0.7	0.8	1.3
				ns	*p<0.05*

Methods S1 Click here to enter text.

(b)

	VvSTS12		VvSTS16		VvSTS18		VvSTS27		VvSTS29	
WBOX Name	M4	101.14	M4	101.14	M4	101.14	M4	101.14	M4	101.14
TTTGACT/C	4	4	8	8	6	4	6	2	2	0
TTGAC	3	3	7	7	7	3	11	4	3	0
TGACT	5	5	10	9	10	5	7	2	3	0
TGACC	7	7	14	13	11	6	9	3	0	0
TGAC	10	12	18	15	14	12	16	10	4	0
TTGACC	0	0	2	2	2	0	2	0	0	0
TTGACT	1	1	2	2	3	1	0	0	1	0
TTTGAC	3	3	4	5	5	3	6	2	1	2
Total	33	35	65	61	58	34	57	23	14	2

(c)

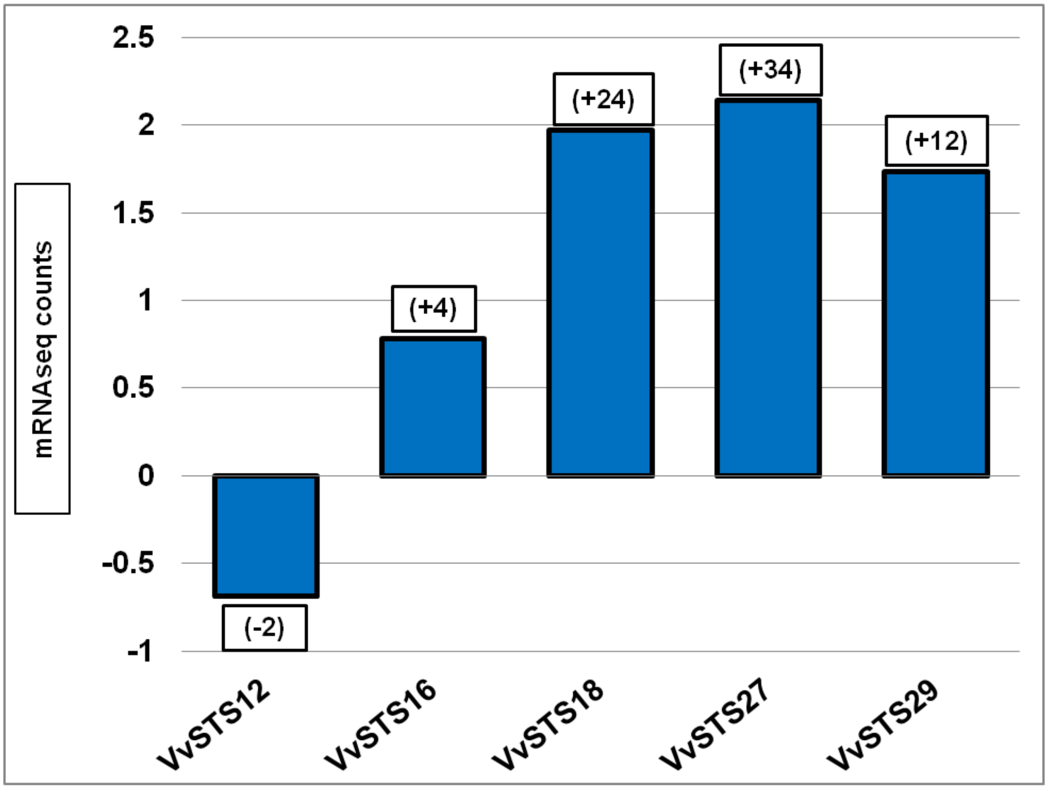

2.5
2
1.5
1
0.5
0
-0.5
-1
mRNAseq counts
(-2)
(+4)
(+24)
(+34)
(+12)
VvSTS12
VvSTS16
VvSTS18
VvSTS27
VvSTS29

(d)

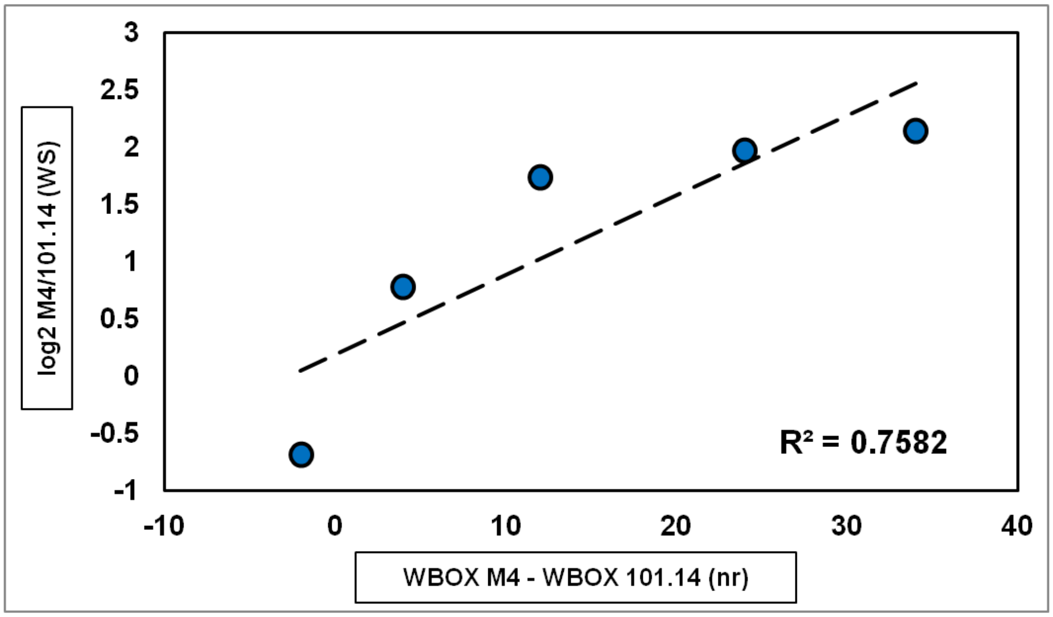

3
2.5
2
1.5
1
0.5
0
-0.5
-1
log2 M4/101.14 (WS)
-10
0
10
20
30
40
WBOX M4 - WBOX 101.14 (nr)
R² = 0.7582

Supplementary Fig. S3 Heat maps showing ABA-related genes in roots and leaves of M4 and 101.14 genotypes**.**

Figure S3. Heat maps with ABA-related genes in roots an leaves of 101.14 and M4. Red and blue represent up- and down-regulated genes upon WS. Expression values are reported as $\log_2$ WS/WW.

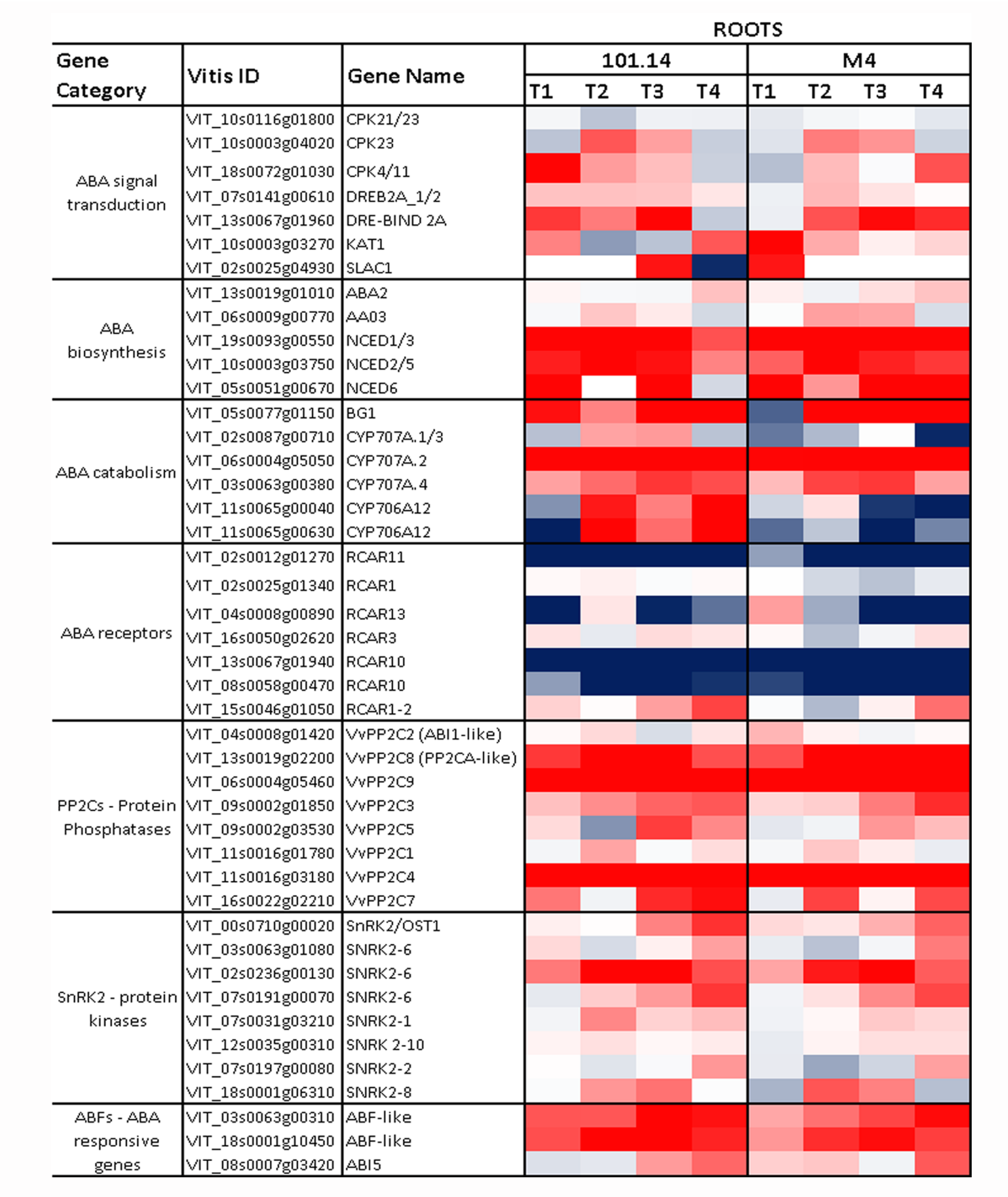

			LEAVES							
Gene Category	Vitis ID	Gene Name	101.14				M4			
			T1	T2	T3	T4	T1	T2	T3	T4
ABA signal transduction	VIT_10s0116g01800	CPK21/23								
	VIT_10s0003g04020	CPK23								
	VIT_18s0072g01030	CPK4/11								
	VIT_07s0141g00610	DREB2A_1/2								
	VIT_13s0067g01960	DRE-BIND 2A								
	VIT_10s0003g03270	KAT1								
	VIT_02s0025g04930	SLAC1								
ABA biosynthesis	VIT_13s0019g01010	ABA2								
	VIT_06s0009g00770	AA03								
	VIT_19s0093g00550	NCED1/3								
	VIT_10s0003g03750	NCED2/5								
	VIT_05s0051g00670	NCED6								
ABA catabolism	VIT_05s0077g01150	BG1								
	VIT_02s0087g00710	CYP707A.1/3								
	VIT_06s0004g05050	CYP707A.2								
	VIT_03s0063g00380	CYP707A.4								
	VIT_11s0065g00040	CYP706A12								
	VIT_11s0065g00630	CYP706A12								
ABA receptors	VIT_02s0012g01270	RCAR11								
	VIT_02s0025g01340	RCAR1								
	VIT_04s0008g00890	RCAR13								
	VIT_16s0050g02620	RCAR3								
	VIT_13s0067g01940	RCAR10								
	VIT_08s0058g00470	RCAR10								
	VIT_15s0046g01050	RCAR1-2								
PP2Cs - Protein Phosphatases	VIT_04s0008g01420	VvPP2C2 (ABI1-like)								
	VIT_13s0019g02200	VvPP2C8 (PP2CA-like)								
	VIT_06s0004g05460	VvPP2C9								
	VIT_09s0002g01850	VvPP2C3								
	VIT_09s0002g03530	VvPP2C5								
	VIT_11s0016g01780	VvPP2C1								
	VIT_11s0016g03180	VvPP2C4								
	VIT_16s0022g02210	VvPP2C7								
SnRK2 - protein kinases	VIT_00s0710g00020	SnRK2/OST1								
	VIT_03s0063g01080	SNRK2-6								
	VIT_02s0236g00130	SNRK2-6								
	VIT_07s0191g00070	SNRK2-6								
	VIT_07s0031g03210	SNRK2-1								
	VIT_12s0035g00310	SNRK 2-10								
	VIT_07s0197g00080	SNRK2-2								
	VIT_18s0001g06310	SNRK2-8								
ABFs - ABA responsive genes	VIT_03s0063g00310	ABF-like								
	VIT_18s0001g10450	ABF-like								
	VIT_08s0007g03420	ABI5								

Supplementary Results S1 M4 and 101.14 genome resequencing.

1. Genomes Resequencing results

The two rootstock genotypes (101.14 and M4) were sequenced by means of a whole genome shotgun approach preparing a "mate paired" library (insert size 1.5 kb) per sample, using as sequencing platform a SOLiD 5500xl by Life Technologies.

1.1 Sequencing depth

For each cultivar approximately 30 Gbp of data have been produced from the "mate paired" libraries (2x60).

	**101.14**	**M4**
Reads	254 173 211	130 210 198
Throughput (Gbp)	30 Gbp	16 Gbp
Raw coverage	68 X	30 X

1.2 Coverage in gene sequences

To assess the mappability of RNA-Seq reads we calculated the number of uniquely mapped reads in each gene using the htseq-count package.

	**101.14**	**M4**
Genes in annotation	29 970	29 970
Genes with 0 unique counts	2 654	2 012
Genes with > 80 unique counts	21 620	21 816
Genes with 0 counts	1	1
Genes with > 80 counts	29 368	29 210

1.3 Variants respect to PN40024

Accepting variants covered at least 20 times, we calculated the average frequency of variants using a sliding window of 2 kb.

	**101.14**	**M4**
N. of Variants	2 233 051	1 671 472
Variants every 2000 bp	9.72 ± 7.11	7.89 ± 7.17
Average distance	217.70 ± 504.37	290.78 ± 731.30

Supplementary Methods S1 mRNA samples preparation and sequencing

Total RNA was extracted from frozen leaves and roots of both genotypes using the "SpectrumTM Plant total RNA Kit" (Sigma) according to manufacturer's instructions. mRNA was purified from the total RNA using the Dynabeads mRNA Direct kit (Invitrogen pn 610.12). A variable quantity of mRNA ranging from 0.4 to 1.6 % respect to the amount total of RNA was obtained. Samples for Ligation Sequencing were prepared according to the "SOLiD Whole transcriptome library preparation" protocol (pn 4452437 Rev.B). The samples were purified before RNase III digestion with Purelink RNA micro kit columns (Invitrogen, pn 12183-016), digested from 3' to 10' depending on the starting amount of mRNA, retro-transcribed, size-selected using Agencourt AMPure XP beads (Beckman Coulter pn A63881) and barcoded during the final amplification. Obtained libraries (an average of 2.5 samples per lane) were sequenced using Applied Biosystems, SOLiDTM 5500XL, which produced paired end reads of 75 and 35 nucleotides for the forward and reverse sequences, respectively. Reads were aligned to the v1 prediction of grapevine PN40024 reference genome (http://genomes.cribi.unipd.it/grape) using PASS aligner (Campagna et al., 2009). The percentage identity was set to 90% and one gap was allowed whereas the quality filtering parameters were set automatically by PASS. A minimum reads length cut-off of 50 and 30 nt was set for the forward sequences and reverse reads, respectively. The spliced reads were identified using the procedure described in PASS manual (http://pass.cribi.unipd.it). The forward and the reverse reads were aligned independently on

the reference genome. The PASS-pair tool of the PASS package was used to perform the pairing between the forward and the reverse reads and to selected only those sequences that were uniquely aligned. Finally htseq- counts program (http://wwwhuber.embl.de/users/anders/HTSeq/doc/count.html) was used to quantify gene abundance.

Supplementary Methods S2 Quantification of Abscisic Acid

Leaf samples (0.5g) were resuspended in 20 ml of a 80% methanol solution containing 100 µg of butylated hydroxytoluene (Sigma Aldrich, St Louis, MO, USA) and 300 ng of d6ABA (OlChemlm, Olomouc, Czech Republic) and incubated overnight at -20°C. After a centrifugation at 5000 rpm for 30 min, 200µl of a 25% ammonia solution were added and the volume of the suspension was reduced to 4 ml by means of a rotavapor under vacuum at 40°C. Once recovered the samples, the pH was reduced through 100 µl of a 2M acetic acid solution and 5 ml of ethyl acetate were added. In order to collect the organic phase, the mixture was centrifuged for 30 min at 2500 rpm and ethyl acetate was removed under vacuum. The dried extract was resuspended by means of 5 ml of a 0.1M acetic acid solution and incubated for 2h at room temperature.

The sample was then loaded on a Sep-Pak® C18 cartridge (Waters, Milford, MA), previously activated with 5 ml of a solution 50% methanol / 50% 0.1M acetic acid. The column was washed with a 17% methanol solution and then the ABA fraction was eluted with 5 ml of a solution 40% methanol / 60% 0.1 M acetic acid. The eluate was pH-adjusted to 8 through a 25% ammonia solution and dried overnight using a SpeedVac at room temperature. The sample was then derivatized with 150 µl of N-Methyl-N-(trimethylsilyl) trifluoroacetamide (MSTFA - Sigma Aldrich, St Louis, MO, USA) for 4h at room temperature under continuous shaking.

Abscisic acid quantification was performed using a GC-MS 7890/5975-MSD (Agilent Technologies, San Jose, CA, USA) injecting 2 µL of the derivatized sample in splitless mode. The column (DB-5, 30 m x 0.25 mm, 0.25 µm, Agilent Technologies), under a constant helium flux of 1ml min^{-1}, was heated 1 min at 70 °C, 6 min ramp to 76 °C, 45 min ramp to 350°C, 1 min at 350 °C, 10 min at 330°C. Ionization was achieved by electron impact at -70 eV and MS Source and

Quad were maintained at 230°C and 150°C, respectively. Spectral acquisition was performed in Selected Ion Monitoring mode (SIM), following the ion relating to d6ABA (m/z = 194; RT = 34.73 min) and ABA (m/z = 190; RT = 34.80 min), setting a dwell time of 20msec. In order to integrate the spectra, chromatograms were analyzed through the software MET-IDEA v. 2.08 (Broeckling et al. 2006).

References

Broeckling CD, Reddy IR, Duran AL, Zhao X, Sumner LW. 2006. MET-IDEA: Data Extraction Tool for Mass Spectrometry-Based Metabolomics. *Analytical Chemistry* **78**:13 4334-41

Supplementary Methods S3. Differential Cluster Analysis (DCA).

Description of the DCA method

Ontology analysis conducted on most representative GO categories allowed us to identify six sub-clusters of DEGs (*i.e.* plant hormones, antioxidant responses, sugars, cell wall, secondary metabolism and transcription factors). Within these sub-clusters, the log_2 (WS/WW) ratio of 101.14 and M4 DEGs of all time points (T1-T4) was retrieved from pairwise statistical analysis and used as data input for the following Differential Clustering Analysis (DCA) conducted on both leaves and roots. The DCA analysis was performed by using an R script, which is a slightly modified version of the original method carried out by Ihmels *et al.* (2005), Lelandais *et al.* (2008) and Cohen *et al.* (2010). The DCA analysis is carried out in three steps that we develop here. (i) The correlation values of all DEGs belonging to the GO categories related to a reference rootstock (rr) and a target rootstock (tr) were initially calculated with the 'cor' R function. (ii) Correlation values of the rr were subsequently clustered by applying the 'kmeans' R function. The number of clusters related to the rr were selected in accordance to an average correlation value, which was heuristically chosen higher than 0.65 for each cluster. The same order chosen for the rr was used to arrange the tr DEGs of the GO-selected categories. Hence, the transcripts from each cluster were co-expressed in the rr (correlation > 0.65) but not necessarily in the tr one. (iii) DEGs related to each cluster of the tr were subsequently grouped into two sub-clusters (a and b) by using a hierarchical clustering method (with 'hclust' R function). The average of the correlation values belonging to each sub-cluster (Ca and Cb) and the average of the correlation

values between the two clusters a and b (Cab) were eventually calculated. Correlation values of tr and rr matrices are graphically represented in white, yellow and red colors for strongly correlated, weakly correlated and anti-correlated genes, respectively. DCA results were finally presented as a unique distance matrix between gene expression measurements in which rr and tr rootstocks were respectively represented in rows and columns. Clusters of each rootstock were compared and assigned to "full", "partial", "split" or "absent" conservation categories after comparing Ca, Cb and Cab values with the threshold T, which is chosen equal to 0.5 in this study. Specifically, if (Ca and Cb) < T the cluster was assigned to the "no conservation" category, if (Ca or Cb) > T the cluster was assigned to the "partial conservation" category, if (Ca and Cb) > T and Cab < T the cluster was assigned to the "split conservation" category and if (Ca, Cb and Cab) > T the cluster was assigned to the "full conservation" category.

Graphical example:

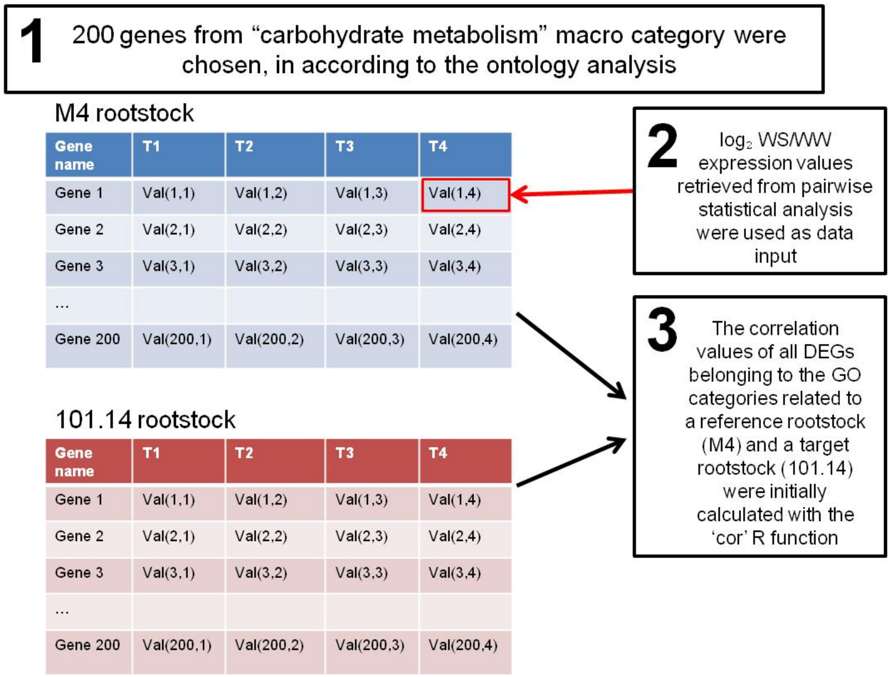

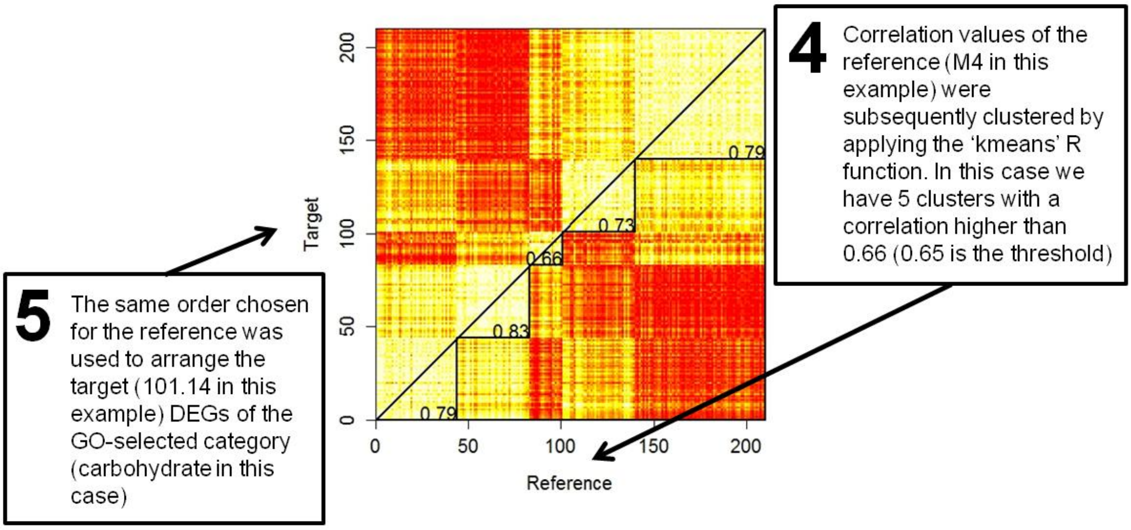

4 Correlation values of the reference (M4 in this example) were subsequently clustered by applying the 'kmeans' R function. In this case we have 5 clusters with a correlation higher than 0.66 (0.65 is the threshold)

5 The same order chosen for the reference was used to arrange the target (101.14 in this example) DEGs of the GO-selected category (carbohydrate in this case)

6 Transcripts from each cluster were co-expressed in the rr (correlation > 0.65) but not necessarily in the tr one

7 DEGs related to each cluster of the target (101.14) were subsequently grouped into two sub-clusters (a and b) by using the 'hclust' R function.

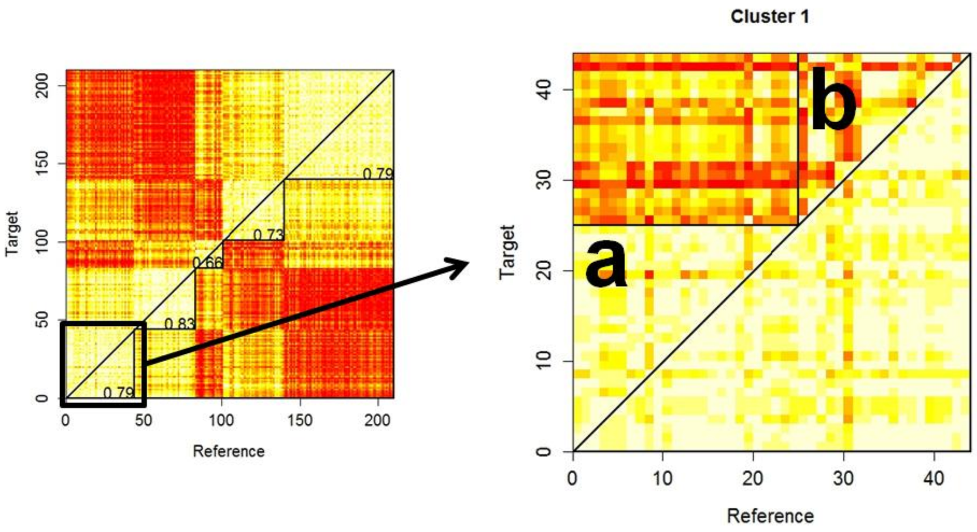

8 If **(Ca and Cb) < T** → **absent** conservation.
If **(Ca or Cb) > T** → **partial** conservation.
If **(Ca and Cb) > T** and **Cab < T** → **split** conservation.
If **(Ca, Cb and Cab) > T** → **full** conservation.

R script for the DCA method

```
#----------------
# Importing data
#----------------
X=read.table("TABLE1.txt",header=TRUE,row.names=1,sep="\t",dec=".")
Y=read.table("TABLE2.txt",header=TRUE,row.names=1,sep="\t",dec=".")

#----------------
# Calculating PCMs
#----------------
XPCM=cor(t(X))
YPCM=cor(t(Y))

#-----------------------------------------------
# Discarding genes with null standard deviation
#-----------------------------------------------
sdX=apply(X,1,sd)
which(sdX==0)
sdY=apply(Y,1,sd)
which(sdY==0)
X2=X[sdX>0&sdY>0,]
Y2=Y[sdX>0&sdY>0,]
dim(X2)
dim(Y2)
XPCM=cor(t(X2))
YPCM=cor(t(Y2))
n=dim(XPCM)[1]
n

#---------------------------------------------------
# How many cluster for the 'kmeans' clusterization?
#---------------------------------------------------
K=10
corValues=vector("list",K)
for (k in 1:K) {
        kmref=kmeans(XPCM,k)
        for (i in 1:k) {
                cori=XPCM[kmref$cluster==i,kmref$cluster==i]
                corValues[[k]]=c(corValues[[k]],mean(cori[upper.tri(cori)]))
        }
}
x11()
boxplot(corValues,xlab="Number of clusters",ylab="Mean correlation values",ylim=c(0,1))
for (r in 1:10) {
        for (k in 1:K) {
                kmref=kmeans(XPCM,k)
                for (i in 1:k) {
                        cori=XPCM[kmref$cluster==i,kmref$cluster==i]
                        corValues[[k]]=c(corValues[[k]],mean(cori[upper.tri(cori)]))
                }
        }
        boxplot(corValues,xlab="Number of clusters",ylab="Mean correlation values",ylim=c(0,1))
}
abline(h=c(0.6,0.7,0.8),lwd=2,lty=2)

#---------------------
# Number of clusters
#---------------------
nClust='n'

#---------------------------------
# Clustering the reference sample
#---------------------------------
kmref=kmeans(XPCM,nClust)
ord=hclust(dist(kmref$centers))$order
kmref$size=kmref$size[ord]
kmref$cluster=kmref$cluster+nClust
for (i in 1:nClust)
        kmref$cluster[kmref$cluster==(ord[i]+nClust)]=i
XPCM1=XPCM[order(kmref$cluster),order(kmref$cluster)]
```

```
YPCM1=YPCM[order(kmref$cluster),order(kmref$cluster)]
ordre=sort(kmref$cluster)
taille=kmref$size
taille
taillec=c(0,cumsum(taille))
taillec
x11()
par(pty="s")
image(0:n,0:n,XPCM1,xlab="Reference",ylab="Target",cex.axis=1.25,cex.lab=1.25)
abline(v=0,h=n,lwd=2)
abline(v=n,h=0,lwd=2)
abline(0,1,lwd=2)
segments(taillec[2:(nClust+1)],taillec[1:nClust],taillec[1:nClust],taillec[1:nClust],lwd=2)
segments(taillec[2:(nClust+1)],taillec[1:nClust],taillec[2:(nClust+1)],taillec[2:(nClust+1)],lwd=
2)

#-------------------------------------------
# Correlation values of reference clusters
#-------------------------------------------
corValues=numeric()
for (i in 1:nClust) {
        cori=XPCM[kmref$cluster==i,kmref$cluster==i]
        corValues=c(corValues,mean(cori[upper.tri(cori)]))
}
corValues

# Adding coorelation values?
text(taillec[1:nClust+1],taillec[1:nClust],round(corValues,2),adj=c(1,0),cex=1.25)

#-----------------------------------------------------------
# Clustering target cluster and calculating Ca, Cb and Cab
#-----------------------------------------------------------
T=0.6 # To choose ?
Ca=vector("numeric",nClust)
Cb=vector("numeric",nClust)
Cab=vector("numeric",nClust)
CaClust=vector("numeric",nClust)
tailleci=0
XPCM2=XPCM1
YPCM2=YPCM1
for (i in 1:nClust) {
        kmtar=kmeans(YPCM1[ordre==i,],2)
        ordrei=1:n
        ordrei[ordre==i]=order(kmtar$cluster)+taillec[i]
        XPCM2=XPCM2[ordrei,ordrei]
        YPCM2=YPCM2[ordrei,ordrei]
        YPCM2reduite=YPCM2[ordre==i,ordre==i]
        tailleci=c(tailleci,max(tailleci)+cumsum(kmtar$size))
        if (kmtar$size[1]>1) {
                C1=YPCM2reduite[1:kmtar$size[1],1:kmtar$size[1]]
                C1=mean(C1[upper.tri(C1)])
        } else C1=1
        if (kmtar$size[2]>1) {

        C2=YPCM2reduite[(kmtar$size[1]+1):sum(kmtar$size),(kmtar$size[1]+1):sum(kmtar$size)]
                C2=mean(C2[upper.tri(C2)])
        } else C2=1
        Cab[i]=mean(YPCM2reduite[1:kmtar$size[1],(kmtar$size[1]+1):sum(kmtar$size)])
        if (C1<C2) {
                Cb[i]=C1
                Ca[i]=C2
                CaClust[i]=2
        } else {
                Cb[i]=C2
                Ca[i]=C1
                CaClust[i]=1
        }
}
Conservation=vector("character",nClust)
Anti.Corr=vector("numeric",nClust)
for (i in 1:nClust) {
```

```
        Conservation[i]=ifelse(Ca[i]>T,ifelse(Cb[i]>T,ifelse(Cab[i]>T,"Full","Split"),"Partial"),
"No")
        Anti.Corr[i]=ifelse(Cab[i]< -T,"Yes","No")
}
Ref.Corr=corValues
res=data.frame(Ref.Corr,CaClust,Ca,Cb,Cab,Conservation,Anti.Corr)
res

#------------------------------------------------
# Graphs of combined matrix and for all clusters
#------------------------------------------------
PCM=XPCM2*lower.tri(XPCM2)+YPCM2*upper.tri(YPCM2)
diag(PCM)=1
x11()
par(pty="s")
image(0:n,0:n,PCM,xlab="Reference",ylab="Target",cex.axis=1.25,cex.lab=1.25)
abline(v=0,h=n,lwd=2)
abline(v=n,h=0,lwd=2)
abline(0,1,lwd=2)
segments(taillec[2:(nClust+1)],taillec[1:nClust],taillec[1:nClust],taillec[1:nClust],lwd=2)
segments(taillec[2:(nClust+1)],taillec[1:nClust],taillec[2:(nClust+1)],taillec[2:(nClust+1)],lwd=
2)
segments(tailleci[2*1:nClust],tailleci[2*1:nClust],tailleci[2*1:nClust],tailleci[2*1:nClust+1],lw
d=2)
segments(tailleci[2*1:nClust],tailleci[2*1:nClust+1],tailleci[2*1:nClust+1],tailleci[2*1:nClust+1
],lwd=2)
segments(tailleci[2*1:nClust-1],tailleci[2*1:nClust-1],tailleci[2*1:nClust-
1],tailleci[2*1:nClust],lwd=2)
segments(tailleci[2*1:nClust-
1],tailleci[2*1:nClust],tailleci[2*1:nClust],tailleci[2*1:nClust],lwd=2)
# For all clusters
for (i in 1:nClust) {
        x11()
        par(pty="s")
        image(taillec[i]:taillec[i+1],taillec[i]:taillec[i+1],PCM[(taillec[i]+1):taillec[i+1],(ta
illec[i]+1):taillec[i+1]],xlab="Reference",ylab="Target",cex.axis=1.25,cex.lab=1.25)
        abline(v=taillec[i],h=taillec[i+1],lwd=2)
        abline(v=taillec[i+1],h=taillec[i],lwd=2)
        abline(0,1,lwd=2)
        segments(taillec[2:(nClust+1)],taillec[1:nClust],taillec[1:nClust],taillec[1:nClust],lwd=
2)
        segments(taillec[2:(nClust+1)],taillec[1:nClust],taillec[2:(nClust+1)],taillec[2:(nClust+
1)],lwd=2)
        segments(tailleci[2*1:nClust],tailleci[2*1:nClust],tailleci[2*1:nClust],tailleci[2*1:nClu
st+1],lwd=2)
        segments(tailleci[2*1:nClust],tailleci[2*1:nClust+1],tailleci[2*1:nClust+1],tailleci[2*1:
nClust+1],lwd=2)
        segments(tailleci[2*1:nClust-1],tailleci[2*1:nClust-1],tailleci[2*1:nClust-
1],tailleci[2*1:nClust],lwd=2)
        segments(tailleci[2*1:nClust-
1],tailleci[2*1:nClust],tailleci[2*1:nClust],tailleci[2*1:nClust],lwd=2)
        title(paste("Cluster",i))
}

#--------------------
# Exporting clusters
#--------------------
DCA.Clusters=data.frame(geneID=dimnames(PCM)[[1]],RefCluster=rep(1:nClust,taille[1:nClust]),TarCl
uster=rep(rep(1:2,nClust),tailleci[1:(2*nClust)+1]-tailleci[1:(2*nClust)]))
write.table(DCA.Clusters,"DCA.Clusters Primary metabolism M4 reference WS
root.txt",sep="\t",row.names=FALSE)
write.table(res,"correlation values Primary metabolism M4 reference WS root.txt")

#---------
# The End
#---------
```
